# Supplementary material for: Synthesis of Novel Arylhydrazones Bearing 8-Trifluoromethyl Quinoline: Crystal Insights, Larvicidal Activity, ADMET Predictions, and Molecular Docking Studies
Source: Pharmaceuticals (Basel). 2025 Nov 26;18(12):1804. doi: 10.3390/ph18121804 (PMC12736387; doi:10.3390/ph18121804)
Supplement: Supplementary file 1 [file pharmaceuticals-18-01804-s001.zip › pharmaceuticals-3975141-supplementary.pdf]

Supplementary Materials

Synthesis of Novel Arylhydrazones Bearing 8-Trifluoromethyl Quinoline: Crystal Insights, Larvicidal Activity, ADMET Predictions, and Molecular Docking Studies

Sukumar Kotyan [1], Shankaranahalli N. Chandana [2], Doddabasavanahalli P. Ganesha [3], Banavase N. Lakshminarayana [4,*], Nefisath Pandikatte [5], Pran Kishore Deb [6], Manik Ghosh [6], Raquel M. Gleiser [7], Mohamad Fawzi Mahomoodally [8,9,10,11,12], Sukainh Aiaysh Alherz [13], Mohamed A. Morsy [13], Hany Ezzat Khalil [13], Mahesh Attimarad [13], Sreeharsha Nagaraja [13], Rashed M. Almuqbil [13], Abdulmalek Ahmed Balgoname [13], Bandar E. Al-Dhubiab [13], Afzal Haq Asif [14], Katharigatta N. Venugopala [13,15,*] and Jagadeesh Prasad Dasappa [1,*]

1 Department of Chemistry, Mangalore University, Mangalagangotri 574199, Karnataka, India; sukumarkotyan8793@gmail.com
2 Department of Physics, Rajeev Institute of Technology, Hassan 573201, Karnataka, India; snc.rit@gmail.com
3 Department of Physics, Malnad College of Engineering, Affiliated to Visvesvaraya Technological University, Hassan 573201, Karnataka, India; ganeshaphysics@gmail.com
4 Research Center, Department of Physics, Adichunchanagiri Institute of Technology, Affiliated to Visvesvaraya Technological University, Jyothinagara, Chikkamagaluru 577102, Karnataka, India
5 Department of PG Studies and Research in Chemistry, Sri Dharmasthala Manjunatheshwara College (Autonomous), Ujire 574240, Karnataka, India; nafeesath@sdmcujire.in
6 Department of Pharmaceutical Sciences and Technology, Birla Institute of Technology (BIT), Mesra 835215, Jharkhand, India; prankishoredeb@bitmesra.ac.in (P.K.D.); manik@bitmesra.ac.in (M.G.)
7 CREAN-IMBIV (UNC-CONICET), Av. Valparaiso s.n., and FCEFyN, Universidad Nacional de Cordoba, Av. V. Sarsfield 299, Cordoba 5000, Argentina; raquel.gleiser@unc.edu.ar
8 Institute of Research and Development, Duy Tan University, Da Nang 550000, Vietnam; mohamadfawzimahomoodally@duytan.edu.vn
9 School of Engineering & Technology, Duy Tan University, Da Nang 550000, Vietnam
10 Laboratory of Natural Products and Medicinal Chemistry (LNPMC), Center for Global Health Research, Saveetha Medical College and Hospital, Saveetha Institute of Medical and Technical Sciences (SIMATS), Thandalam, Chennai 602105, Tamil Nadu, India
11 Centre of Excellence for Pharmaceutical Sciences (Pharmacen), North West University, Potchefstroom 2520, South Africa
12 Department of Health Sciences, Faculty of Medicine and Health Sciences, University of Mauritius, Reduit 80837, Mauritius
13 Department of Pharmaceutical Sciences, College of Clinical Pharmacy, King Faisal University, Al-Ahsa 31982, Saudi Arabia; salharz@kfu.edu.sa (S.A.A.); momorsy@kfu.edu.sa (M.A.M.); heahmed@kfu.edu.sa (H.E.K.); mattimarad@kfu.edu.sa (M.A.); sharsha@kfu.edu.sa (S.N.); ralmuqbil@kfu.edu.sa (R.M.A.); abalgoname@kfu.edu.sa (A.A.B.); baldhubiab@kfu.edu.sa (B.E.A.-D.)
14 Department of Pharmacy Practice, College of Clinical Pharmacy, King Faisal University, Al-Ahsa 31982, Saudi Arabia; ahasif@kfu.edu.sa
15 Department of Biotechnology and Food Science, Faculty of Applied Sciences, Durban University of Technology, Durban 4001, South Africa
* Correspondence: bnlphysics@gmail.com (B.N.L.); kvenugopala@kfu.edu.sa (K.N.V.); jprasad2003@gmail.com (J.P.D.)

TABLE OF CONTENTS

Sl No	Description	Page No.
1.	**Figure S1.** ^{1}H NMR spectrum of (*E*)-4-(2-(3-bromo-4-fluorobenzylidene) hydrazinyl) -8-(trifluoromethyl)quinoline **(6a)**	4
2.	**Figure S2.** ^{19}F NMR spectrum of (*E*)-4-(2-(3-bromo-4-fluorobenzylidene) hydrazinyl) -8-(trifluoromethyl)quinoline **(6a)**	5
3.	**Figure S3.** ^{13}C NMR Spectrum of (*E*)-4-(2-(3-bromo-4-fluorobenzylidene) hydrazinyl)-8-(trifluoromethyl)quinoline **(6a)**	6
4.	**Figure S4.** HRMS of (*E*)-4-(2-(3-bromo-4-fluorobenzylidene)hydrazinyl)-8-(trifluoromethyl)quinoline **(6a)**	7
5.	**Figure S5.** ^{1}H NMR spectrum of (*E*)-4-(2-(5-bromo-2-methoxybenzylidene) hydrazinyl)-8-(trifluoromethyl)quinoline (**6b**)	8
6.	**Figure S6.** ^{19}F NMR spectrum of (*E*)-4-(2-(5-bromo-2-methoxybenzylidene) hydrazinyl)-8-(trifluoromethyl)quinoline (**6b**)	9
7.	**Figure S7.** ^{13}CNMR Spectrum of (*E*)-4-(2-(5-bromo-2-methoxybenzylidene) hydrazinyl)-8-(trifluoromethyl)quinoline (**6b**)	10
8.	**Figure S8.** HRMS of (*E*)-4-(2-(5-bromo-2-methoxybenzylidene)hydrazinyl)-8-(trifluoromethyl)quinoline (**6b**)	11
9.	**Figure S9.** ^{1}H NMR spectrum of (*E*)-4-(2-(4,5-dimethoxy-2-nitrobenzylidene) hydrazinyl)-8-(trifluoromethyl)quinoline (**6c**)	12
10.	**Figure S10.** ^{19}F NMR spectrum of (*E*)-4-(2-(4,5-dimethoxy-2-nitrobenzylidene) hydrazinyl)-8-(trifluoromethyl)quinoline (**6c**)	13
11.	**Figure S11.** ^{13}CNMR Spectrum of (*E*)-4-(2-(4,5-dimethoxy-2-nitrobenzylidene) hydrazinyl)-8-(trifluoromethyl)quinoline (**6c**)	14
12.	**Figure S12.** HRMS of (*E*)-4-(2-(4,5-dimethoxy-2-nitrobenzylidene) hydrazinyl)-8-(trifluoromethyl)quinoline (**6c**)	15
13.	**Figure S13.** ^{1}H NMR spectrum of (*E*)-4-(2-(3-bromo-4-methoxybenzylidene) hydrazinyl)-8-(trifluoromethyl)quinoline (**6d**)	16
14.	**Figure S14.** ^{19}F NMR spectrum of (*E*)-4-(2-(3-bromo-4-methoxybenzylidene) hydrazinyl)-8-(trifluoromethyl)quinoline (**6d**)	17
15.	**Figure S15.** ^{13}CNMR Spectrum of (*E*)-4-(2-(3-bromo-4-methoxybenzylidene) hydrazinyl)-8-(trifluoromethyl)quinoline (**6d**)	18
16.	**Figure S16.** HRMS of (*E*)-4-(2-(3-bromo-4-methoxybenzylidene)hydrazinyl)-8-(trifluoromethyl)quinoline **(6d)**	19
17.	**Figure S17.** ^{1}H NMR spectrum of (*E*)-4-(2-(2-chloro-5-nitrobenzylidene) hydrazinyl) -8-(trifluoromethyl)quinoline (**6e**)	20
18.	**Figure S18.** ^{19}F NMR spectrum of (*E*)-4-(2-(2-chloro-5-nitrobenzylidene) hydrazinyl) -8-(trifluoromethyl)quinoline (**6e**)	21
19.	**Figure S19.** ^{13}CNMR Spectrum of (*E*)-4-(2-(2-chloro-5-nitrobenzylidene) hydrazinyl)-8-(trifluoromethyl)quinoline (**6e**)	22
20.	**Figure S20.** HRMS (*E*)-4-(2-(2-chloro-5-nitrobenzylidene)hydrazinyl)-8-(trifluoromethyl) quinoline (**6e**)	23

21.	**Figure S21.** ^{1}H NMR spectrum of (*E*)-4-(2-(4-methoxy-3-nitrobenzylidene) hydrazinyl)-8-(trifluoromethyl)quinoline (**6f**)	24
22.	**Figure S22.** ^{19}F NMR spectrum of (*E*)-4-(2-(4-methoxy-3-nitrobenzylidene) hydrazinyl)-8-(trifluoromethyl)quinoline (**6f**)	25
23.	**Figure S23.** ^{13}CNMR Spectrum of (*E*)-4-(2-(4-methoxy-3-nitrobenzylidene) hydrazinyl)-8-(trifluoromethyl)quinoline (**6f**)	26
24.	**Figure S24.** HRMS of (*E*)-4-(2-(4-methoxy-3-nitrobenzylidene)hydrazinyl)-8-(trifluoromethyl)quinoline (**6f**)	27
25.	**Figure S25.** ^{1}H NMR spectrum of (*E*)-4-(2-(2,4-dimethoxybenzylidene) hydrazinyl)-8-(trifluoromethyl)quinoline (**6g**)	28
26.	**Figure S26.** ^{19}F NMR spectrum of (*E*)-4-(2-(2,4-dimethoxybenzylidene) hydrazinyl)-8-(trifluoromethyl)quinoline (**6g**)	29
27.	**Figure S27.** ^{13}CNMR Spectrum of (*E*)-4-(2-(2,4-dimethoxybenzylidene) hydrazinyl)-8-(trifluoromethyl)quinoline (**6g**)	30
28.	**Figure S28.** HRMS of (*E*)-4-(2-(2,4-dimethoxybenzylidene)hydrazinyl)-8-(trifluoromethyl)quinoline (**6g**)	31
29.	**Figure S29.** ^{1}H NMR spectrum of (*E*)-4-(2-(4-fluorobenzylidene)hydrazinyl)-8-(trifluoromethyl)quinoline (**6h**)	32
30.	**Figure S30.** ^{19}F NMR spectrum of (*E*)-4-(2-(4-fluorobenzylidene)hydrazinyl)-8-(trifluoromethyl)quinoline (**6h**)	33
31.	**Figure S31.** ^{13}CNMR Spectrum of (*E*)-4-(2-(4-fluorobenzylidene)hydrazinyl)-8-(trifluoromethyl)quinoline (**6h**)	34
32.	**Figure S32.** HRMS of (*E*)-4-(2-(4-fluorobenzylidene)hydrazinyl)-8-(trifluoromethyl)quinoline (**6h**)	35
33.	**Figure S33.** ^{1}H NMR spectrum of (*E*)-4-(2-(4-bromobenzylidene)hydrazinyl)-8-(trifluoromethyl)quinoline (**6i**)	36
34.	**Figure S34.** ^{19}F NMR spectrum of (*E*)-4-(2-(4-bromobenzylidene)hydrazinyl)-8-(trifluoromethyl)quinoline (**6i**)	37
35.	**Figure S35.** ^{13}CNMR Spectrum of (*E*)-4-(2-(4-bromobenzylidene)hydrazinyl)-8-(trifluoromethyl)quinoline (**6i**)	38
36.	**Figure S36.** HRMS of (*E*)-4-(2-(4-bromobenzylidene)hydrazinyl)-8-(trifluoromethyl)quinoline (**6i**)	39
37.	**Figure S37.** Check CIF report on (*E*)-4-(2-(3-bromo-4-methoxybenzylidene)hydrazinyl)-8-(trifluoromethyl)quinoline (**6d**)	40-43
38.	Table S1. Tabular presentation of in silico ADME parameters for all compounds (**6a-6i**) under study.	44

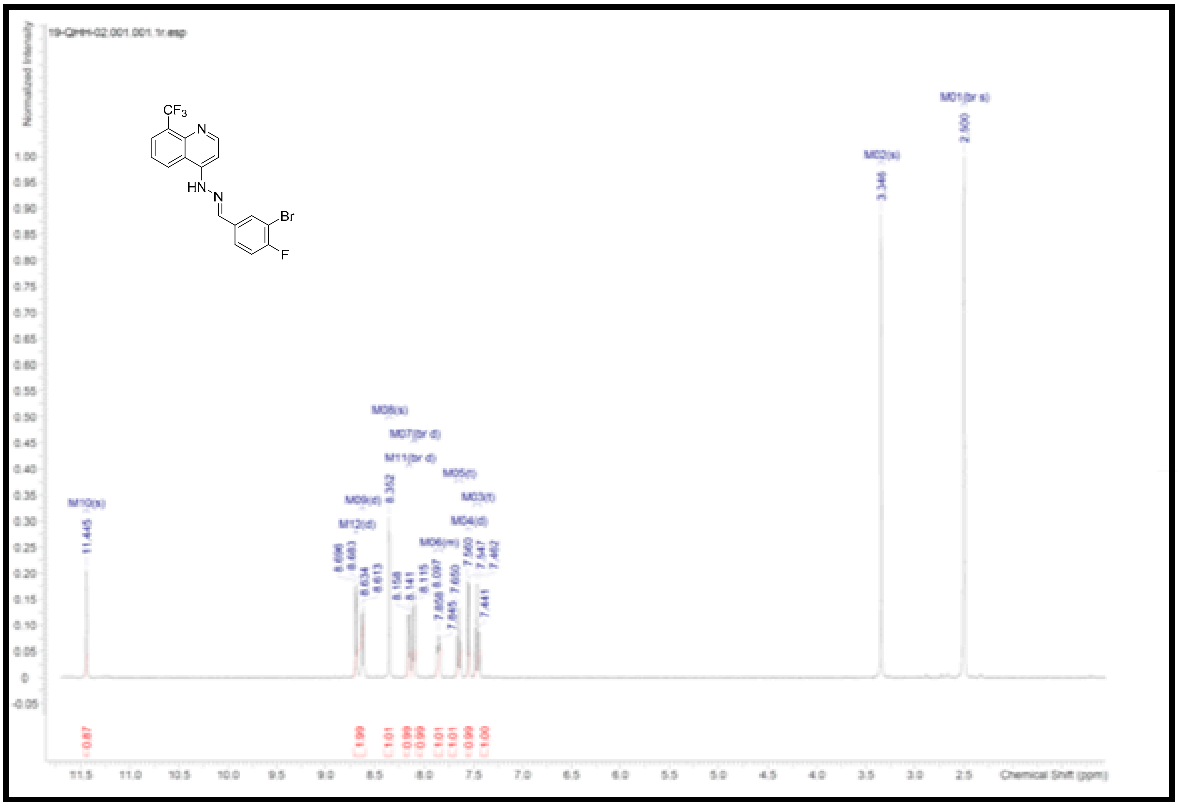

Figure S1. ^{1}H NMR spectrum of (*E*)-4-(2-(3-bromo-4-fluorobenzylidene)hydrazinyl)-8-(trifluoromethyl)quinoline (**6a**).

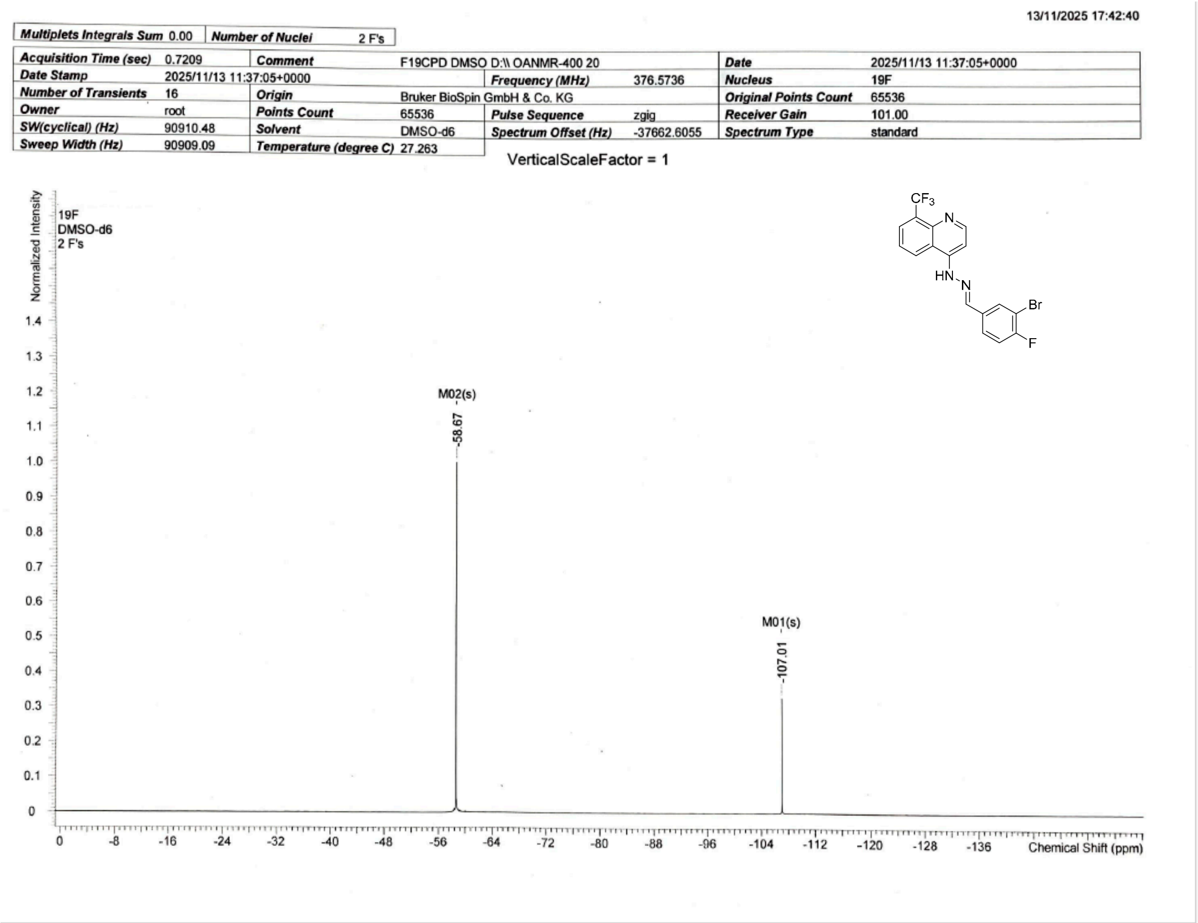

Figure S2. ^{19}F NMR spectrum of (*E*)-4-(2-(3-bromo-4-fluorobenzylidene) hydrazinyl) -8-(trifluoromethyl)quinoline **(6a).**

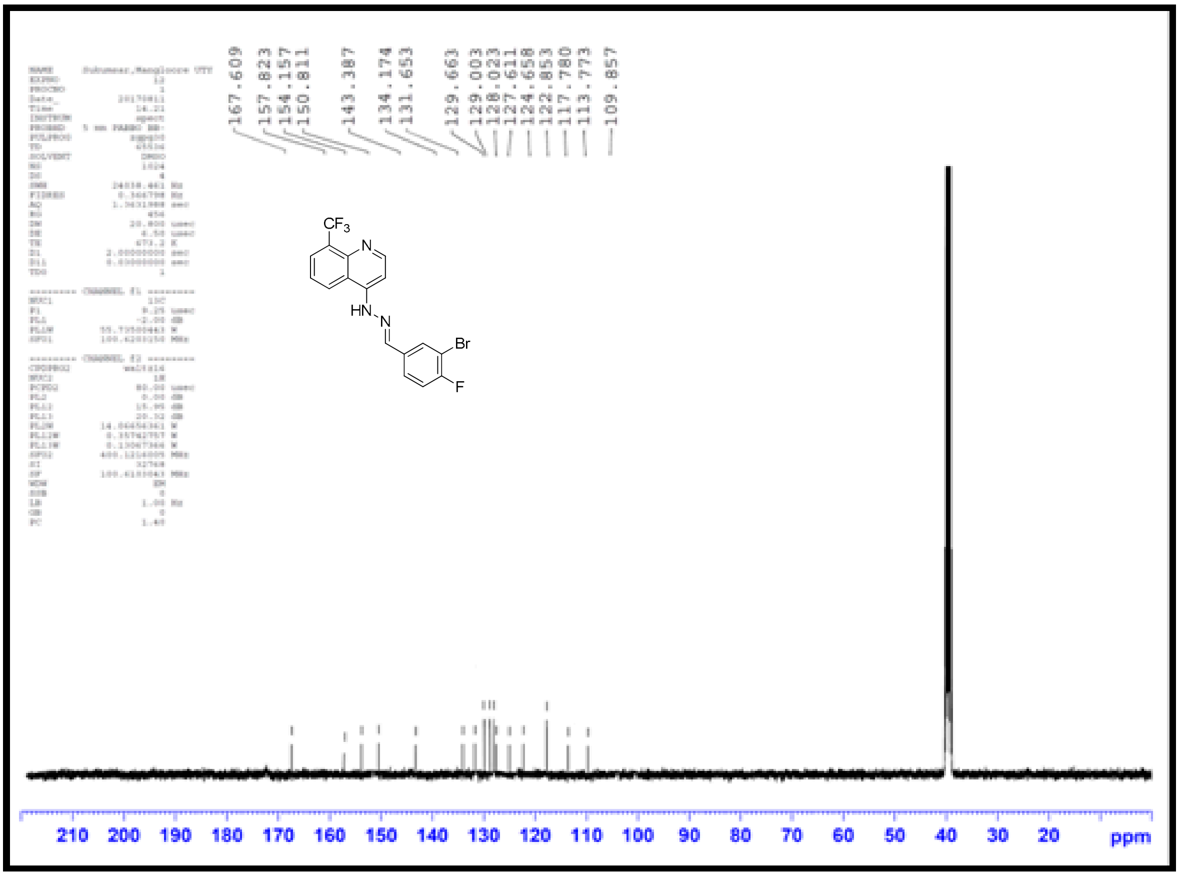

Figure S3. ^{13}C NMR Spectrum of (*E*)-4-(2-(3-bromo-4-fluorobenzylidene)hydrazinyl)-8-(trifluoromethyl)quinoline (**6a**).

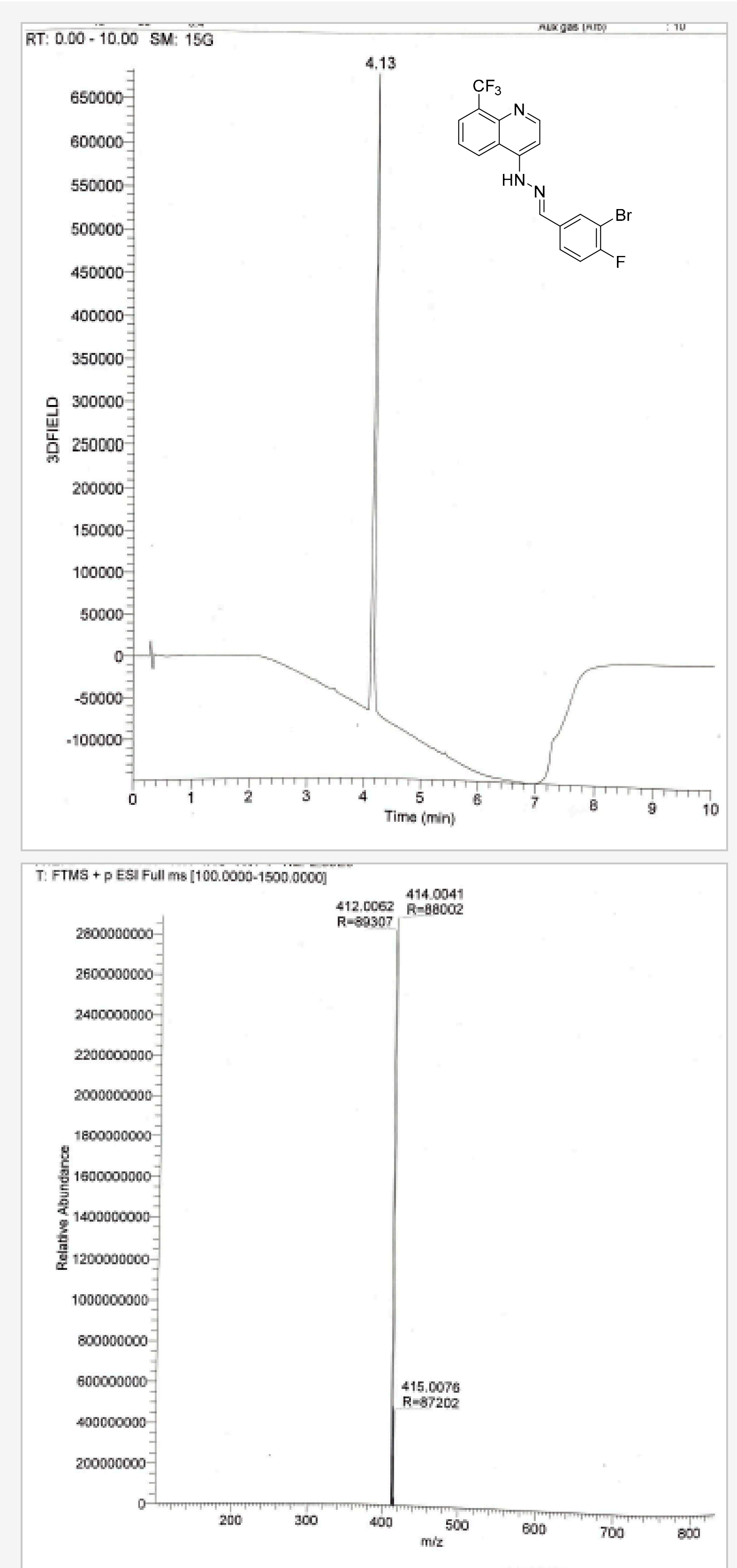

Figure S4. HRMS of (*E*)-4-(2-(3-bromo-4-fluorobenzylidene)hydrazinyl)-8-(trifluoromethyl)quinoline (**6a**).

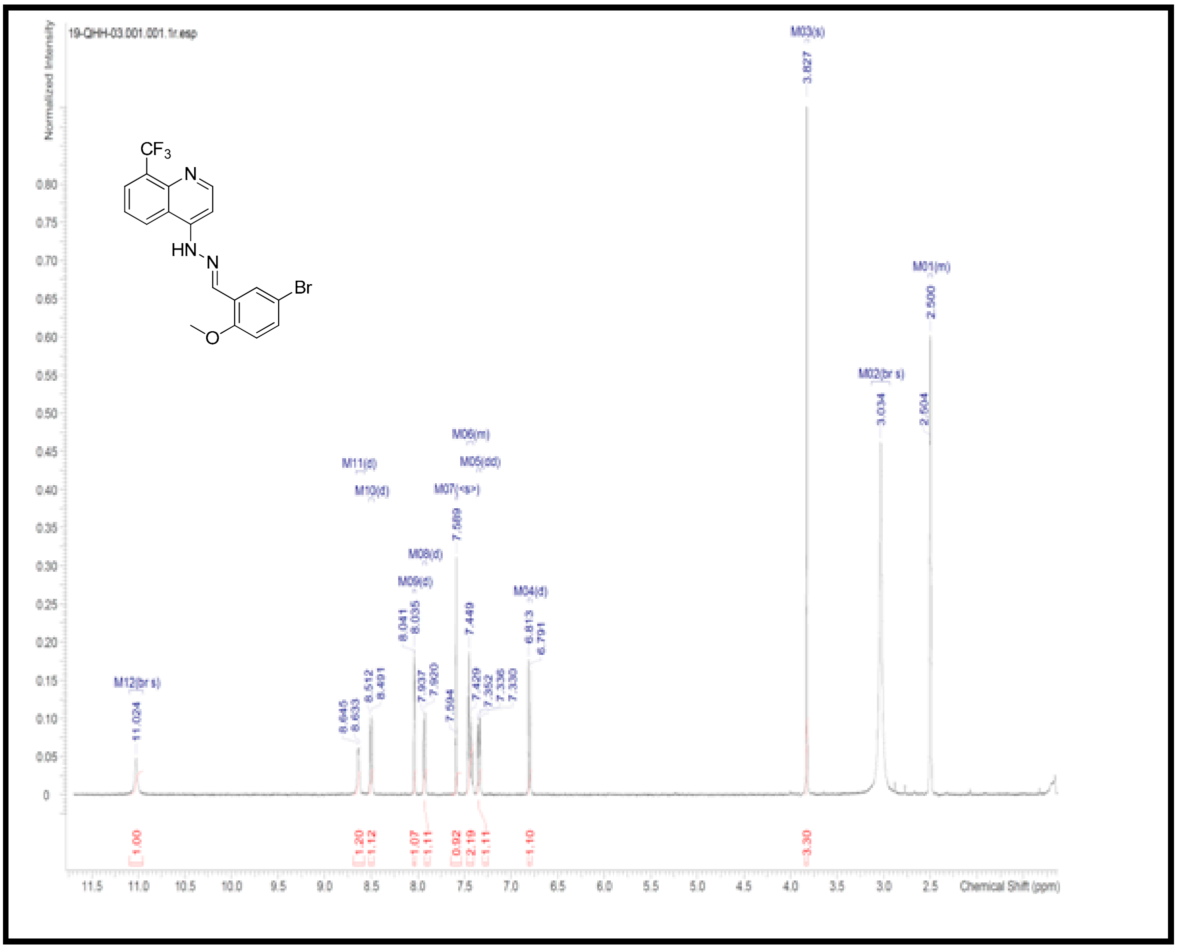

Figure S5. ^{1}H NMR spectrum of (*E*)-4-(2-(5-bromo-2-methoxybenzylidene)hydrazinyl)-8-(trifluoromethyl)quinoline (**6b**).

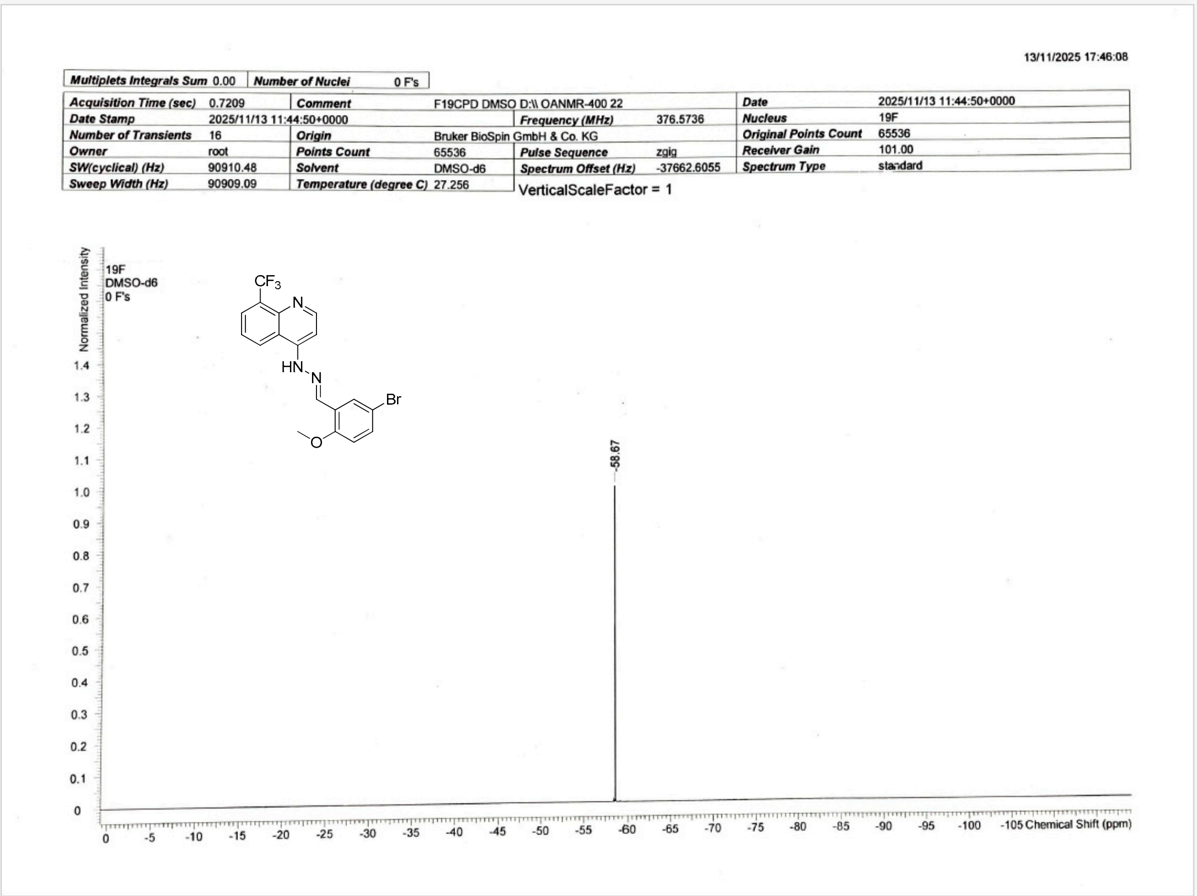

Figure S6. ^{19}F NMR spectrum of (*E*)-4-(2-(5-bromo-2-methoxybenzylidene) hydrazinyl)-8-(trifluoromethyl)quinoline (**6b**).

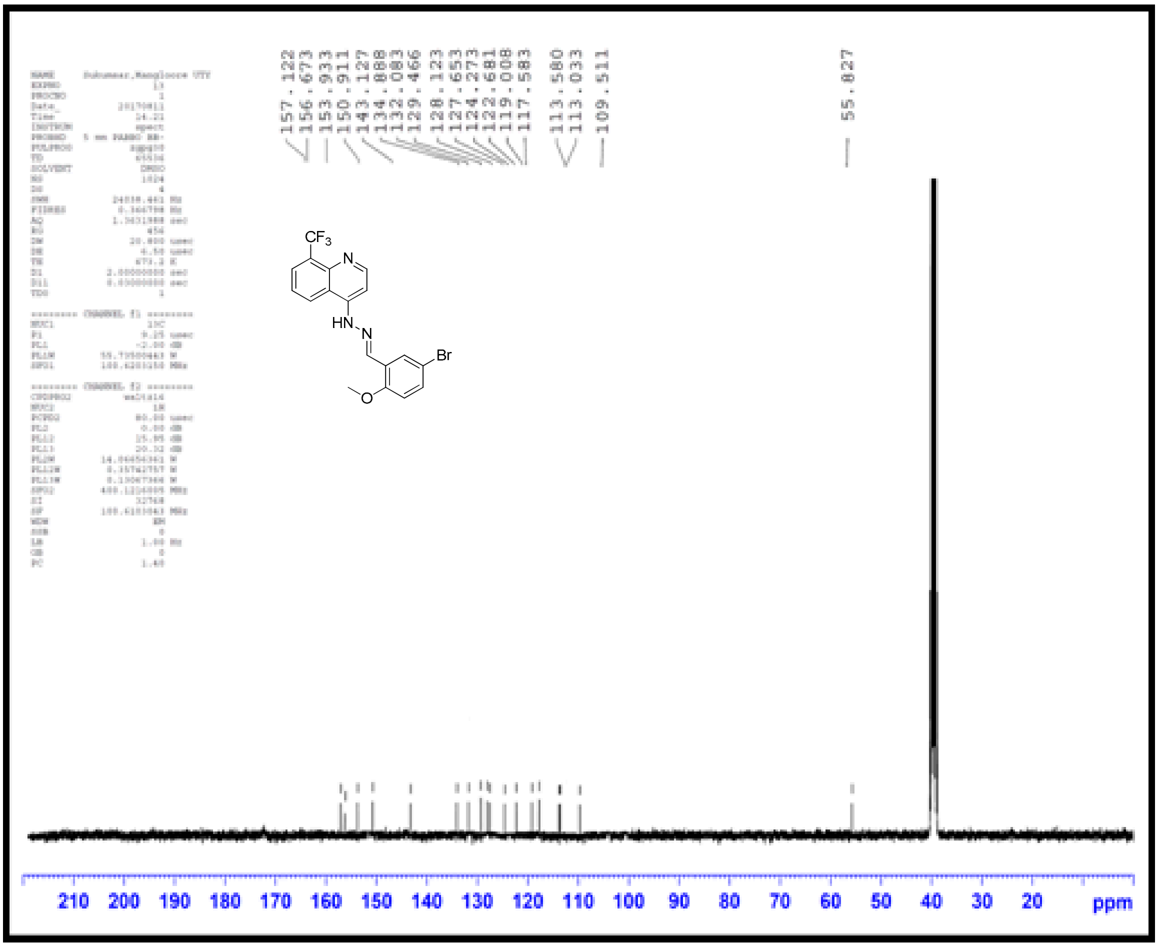

Figure S7. ^{13}CNMR spectrum of (*E*)-4-(2-(5-bromo-2-methoxybenzylidene)hydrazinyl)-8-(trifluoromethyl)quinoline (**6b**).

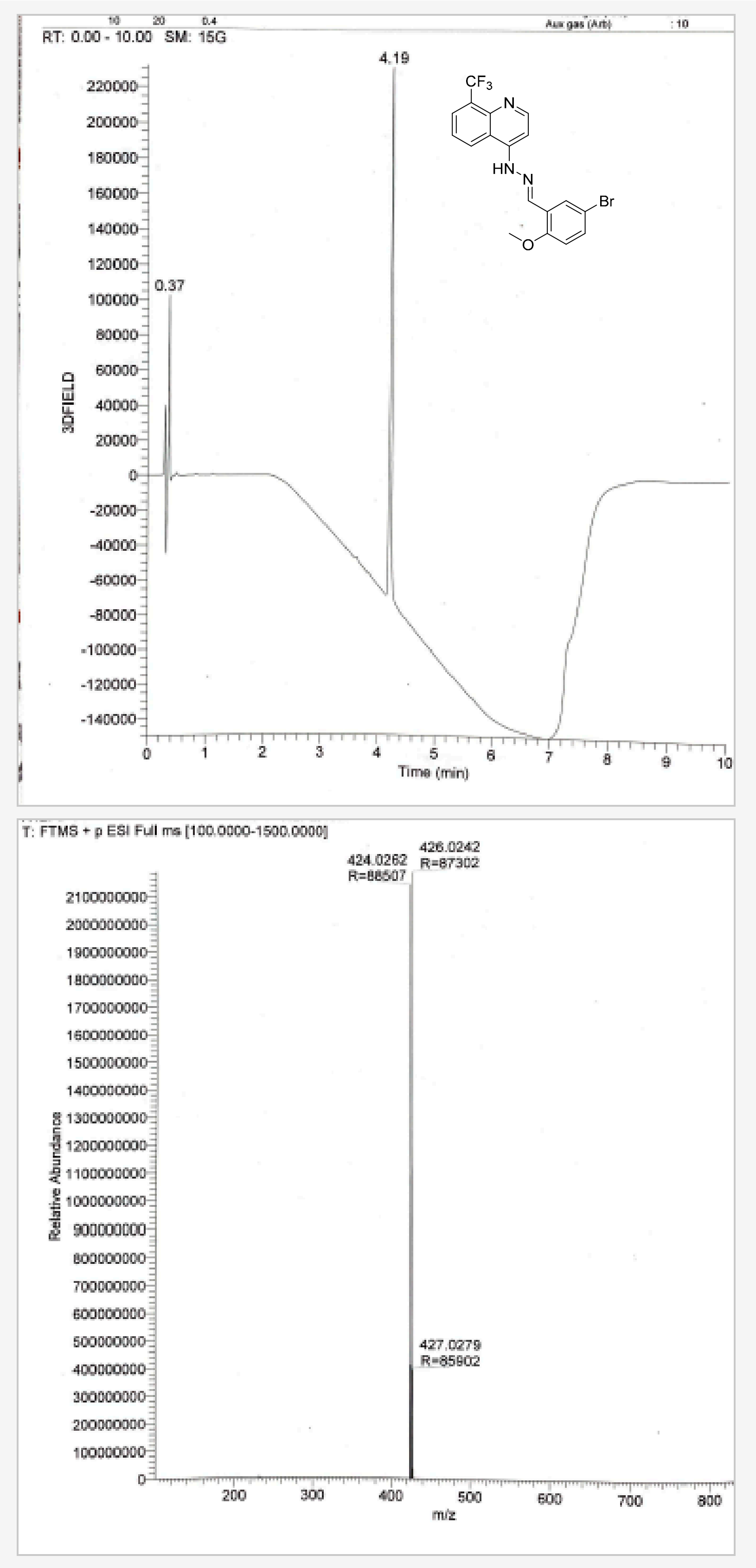

Figure S8. HRMS of (*E*)-4-(2-(5-bromo-2-methoxybenzylidene)hydrazinyl)-8-(trifluoromethyl)quinoline (**6b**).

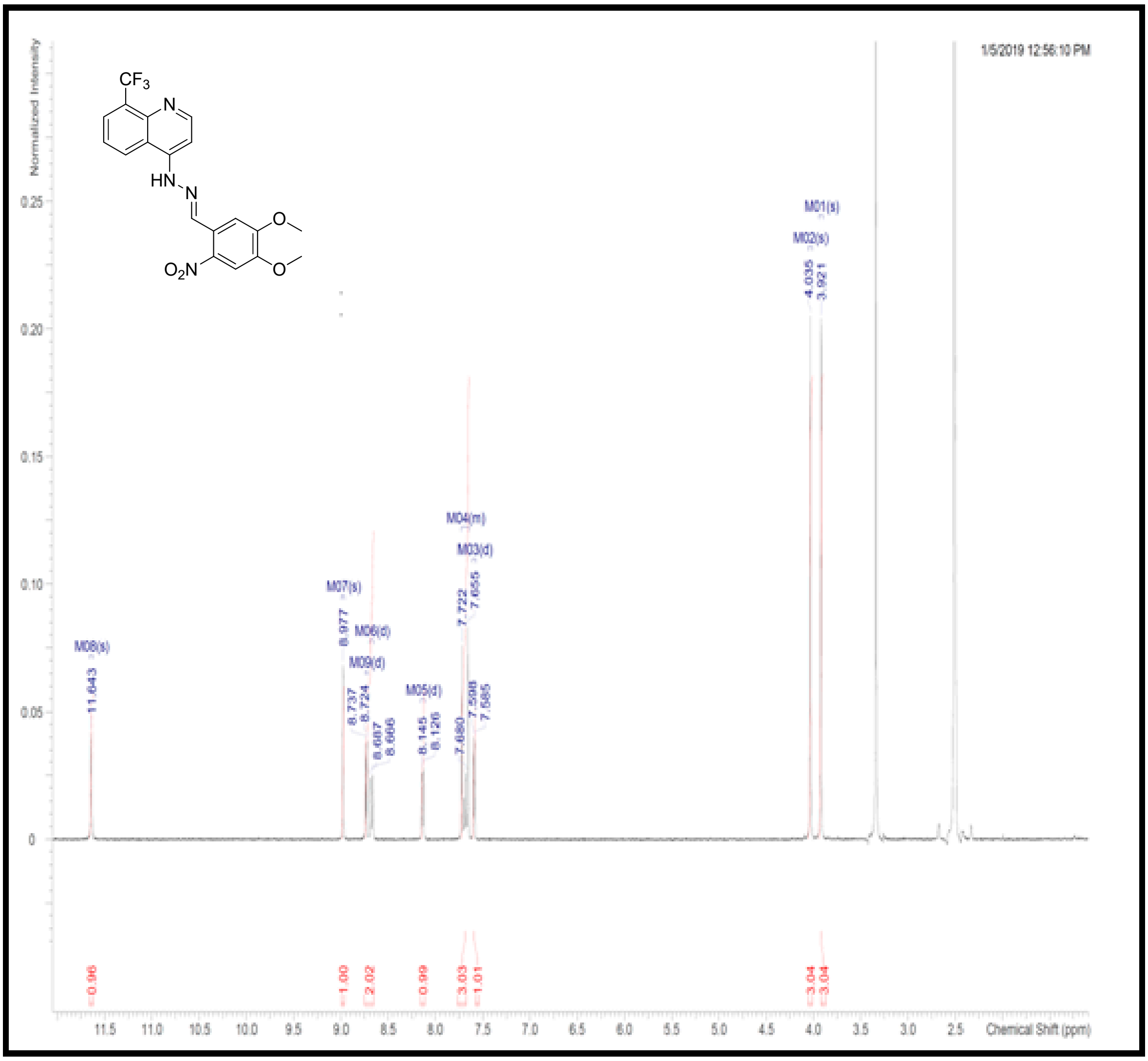

Figure S9. ^{1}H NMR spectrum of (*E*)-4-(2-(4,5-dimethoxy-2-nitrobenzylidene)hydrazinyl)-8-(trifluoromethyl)quinoline (**6c**).

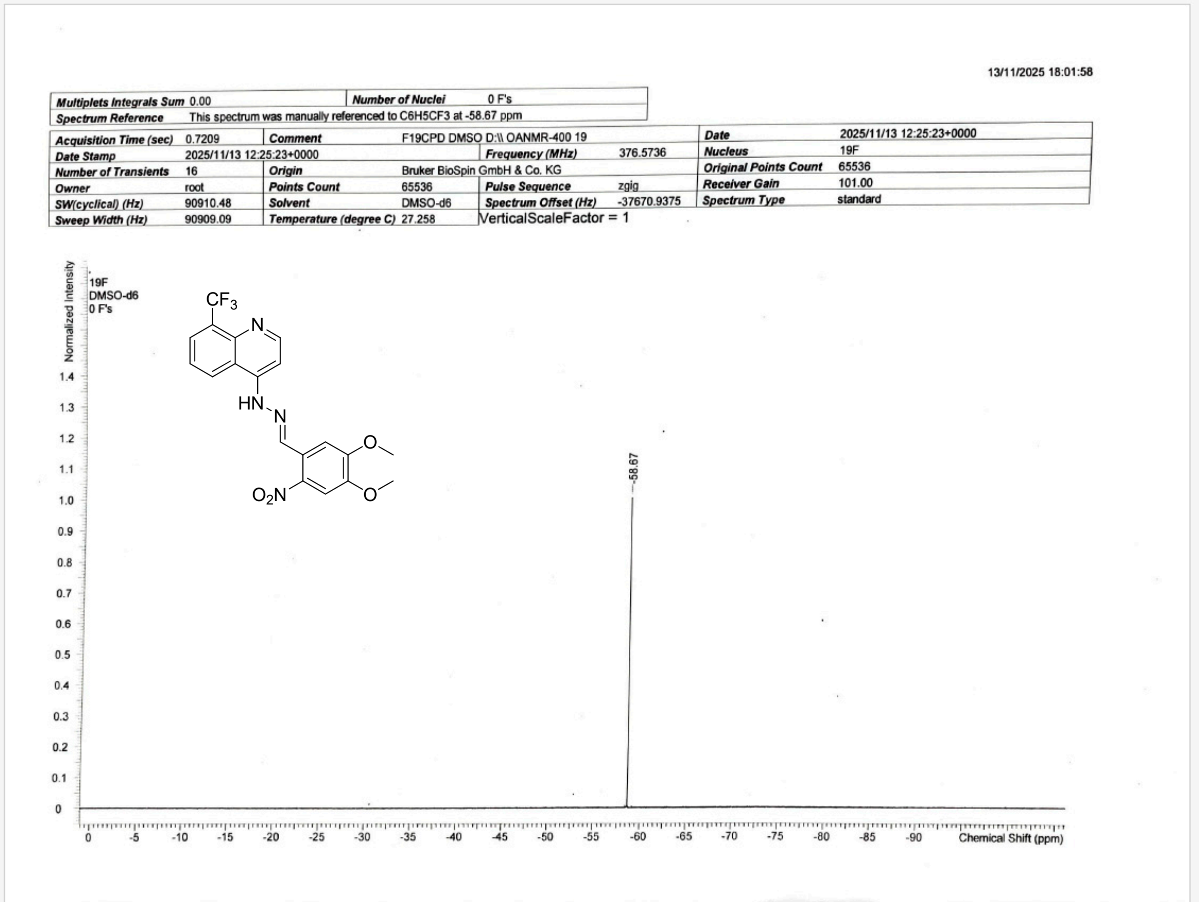

Figure S10. 19F NMR spectrum of (*E*)-4-(2-(4,5-dimethoxy-2-nitrobenzylidene) hydrazinyl)-8-(trifluoromethyl)quinoline (**6c**).

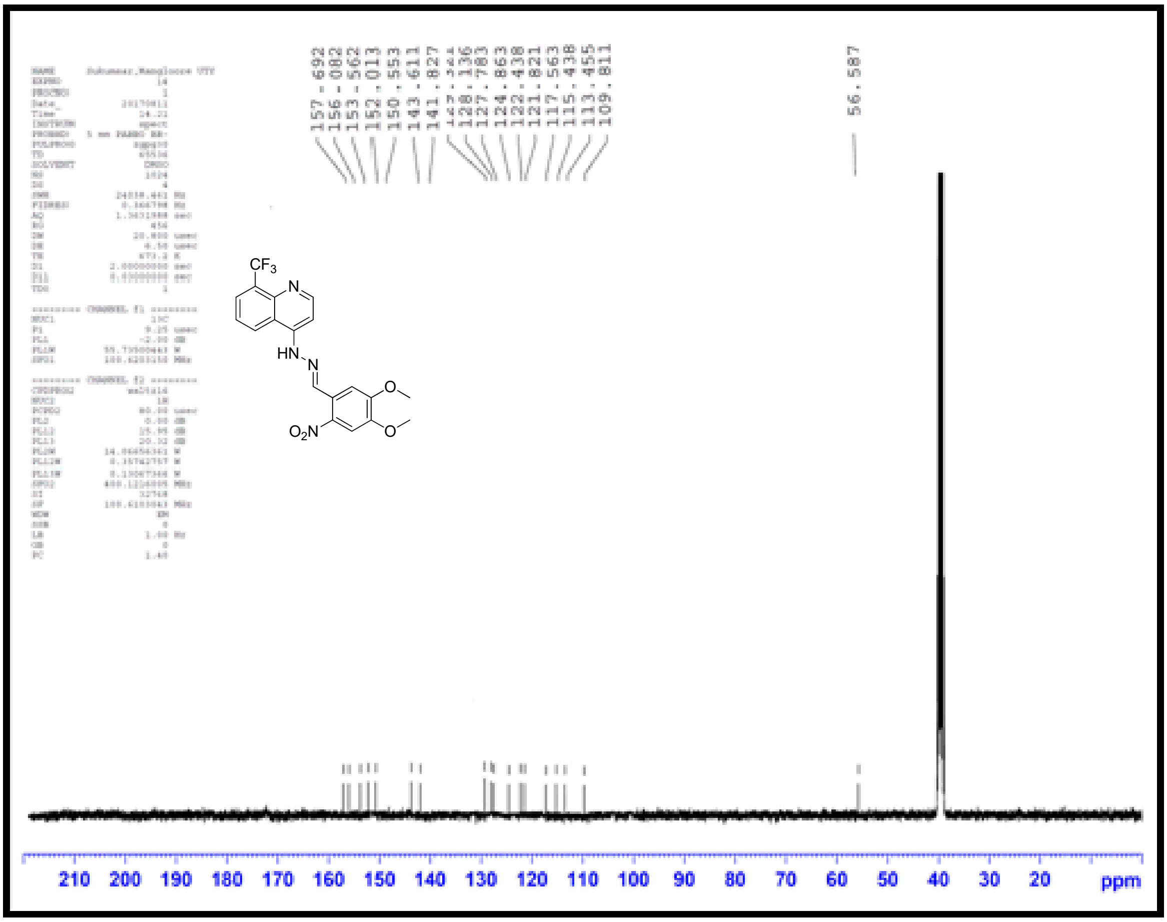

Figure S11. ^{13}C NMR spectrum of (*E*)-4-(2-(4,5-dimethoxy-2-nitrobenzylidene)hydrazinyl)-8-(trifluoromethyl)quinoline (**6c**).

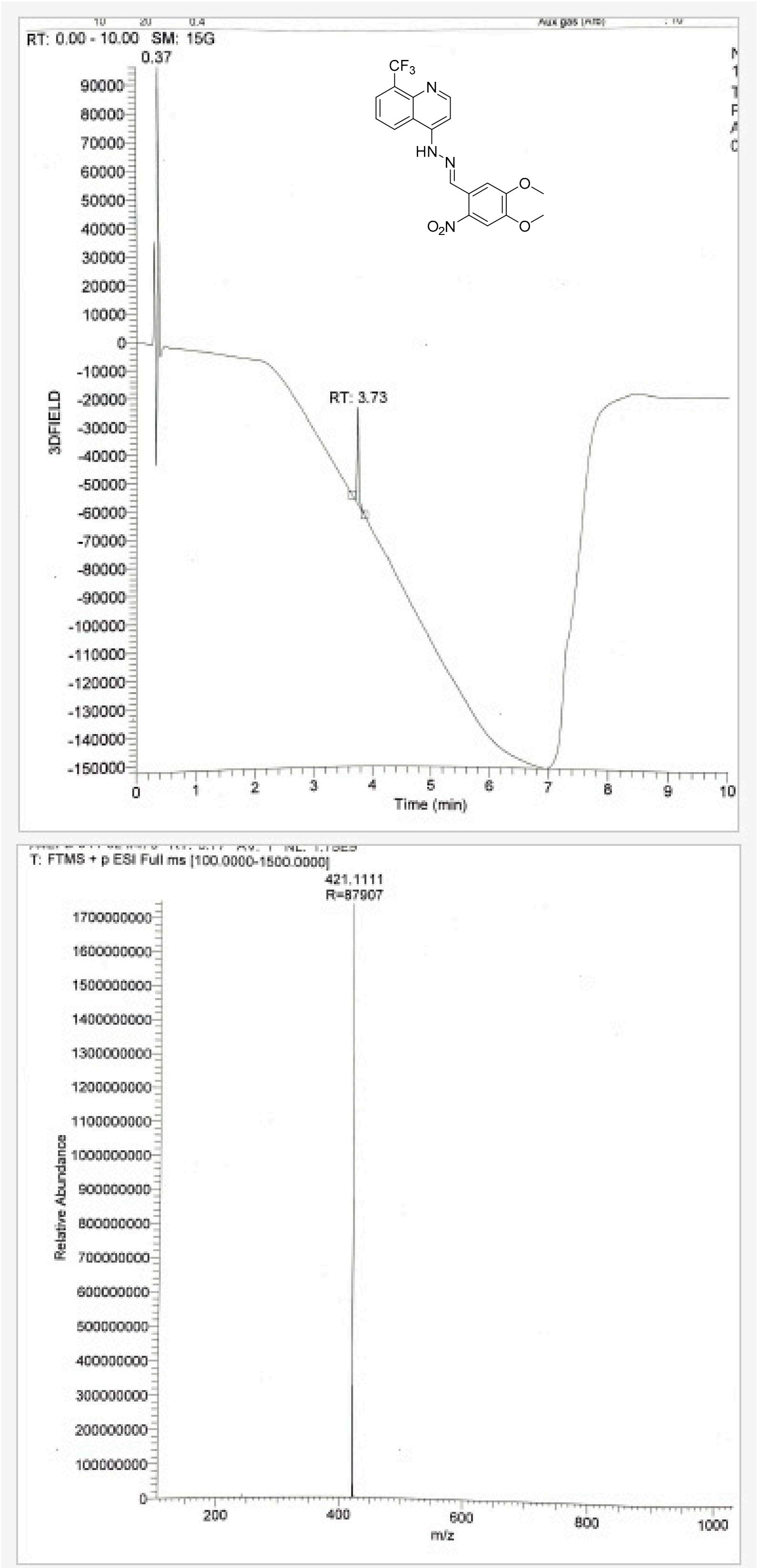

Figure S12. HRMS of (*E*)-4-(2-(4,5-dimethoxy-2-nitrobenzylidene)hydrazinyl)-8-(trifluoromethyl)quinoline (**6c**).

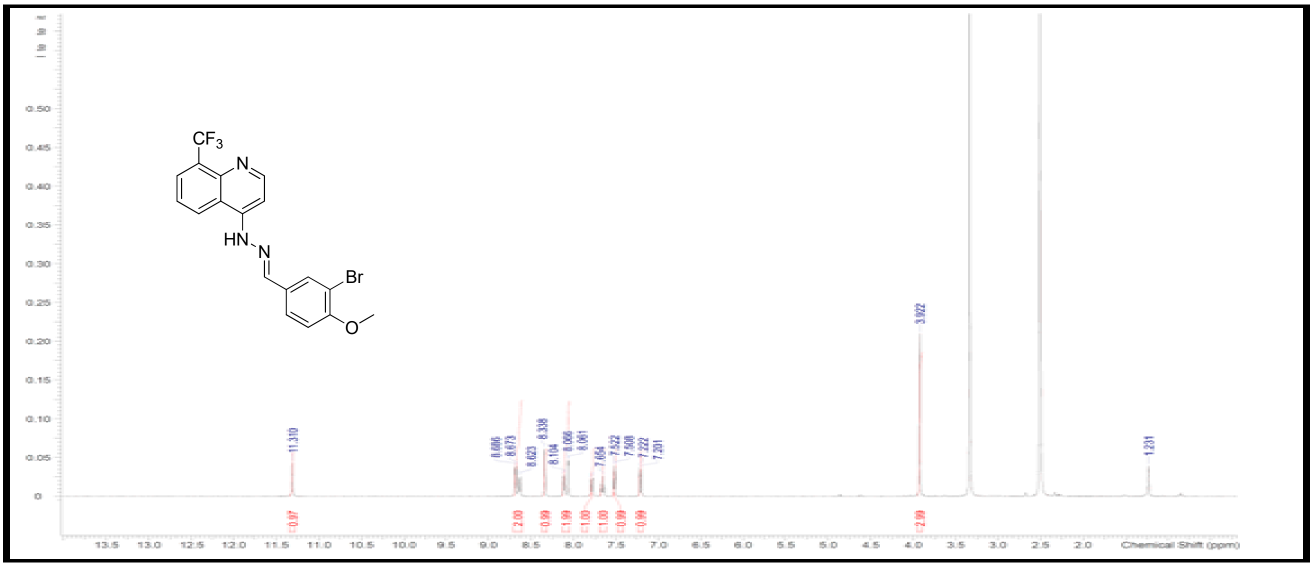

Figure S13. ^{1}H NMR spectrum of (*E*)-4-(2-(3-bromo-4-methoxybenzylidene)hydrazinyl)-8-(trifluoromethyl)quinoline (**6d**).

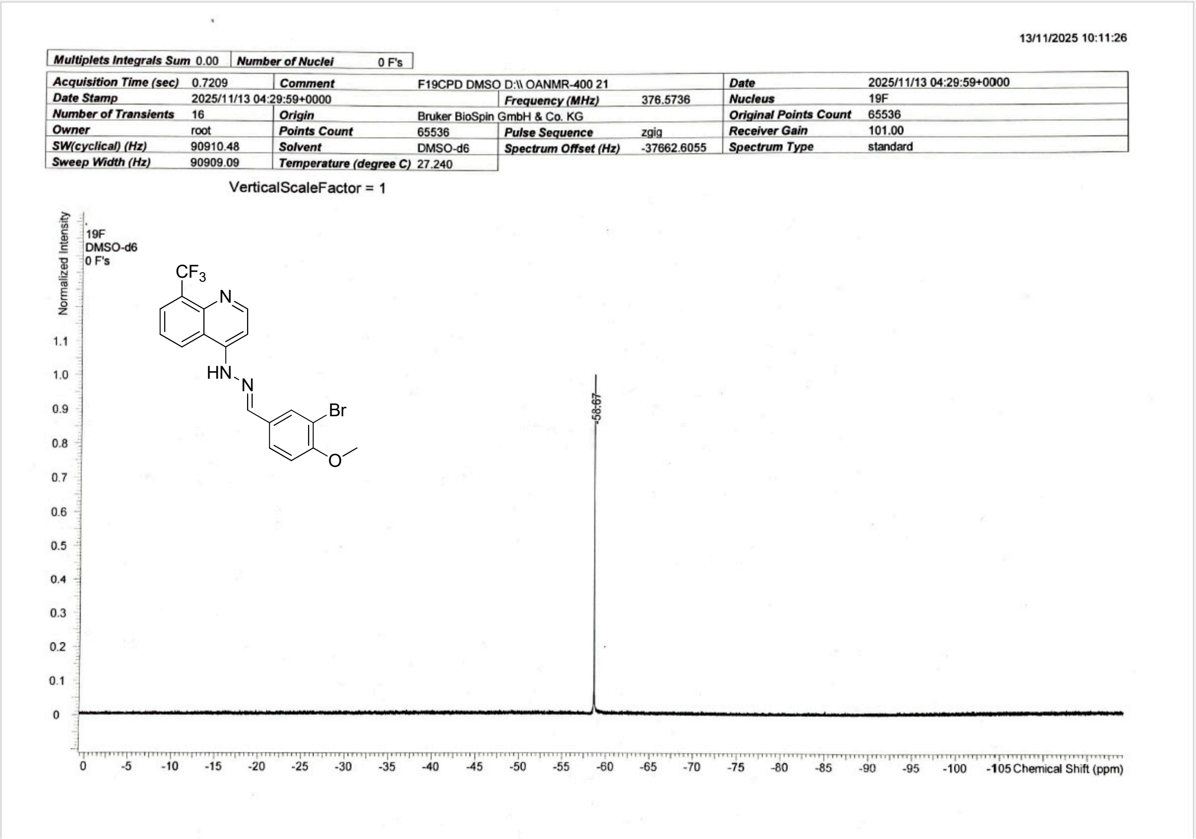

Figure S14. ^{19}F NMR spectrum of (E)-4-(2-(3-bromo-4-methoxybenzylidene) hydrazinyl)-8-(trifluoromethyl)quinoline **(6d).**

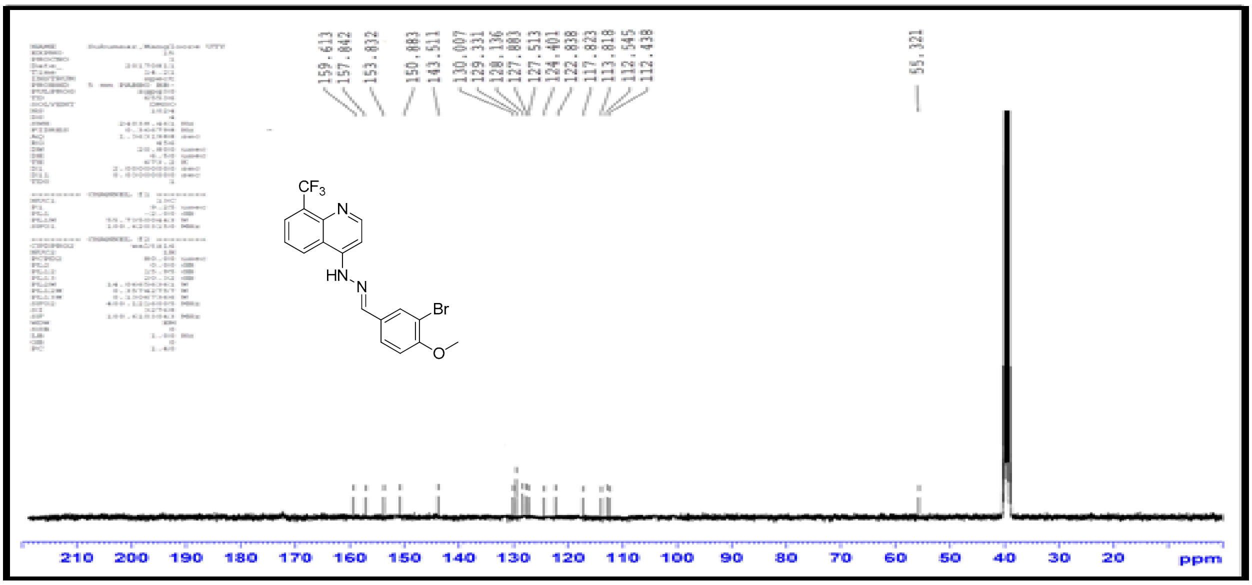

Figure S15. ^{13}C NMR spectrum of (*E*)-4-(2-(3-bromo-4-methoxybenzylidene)hydrazinyl)-8-(trifluoromethyl)quinoline (**6d**).

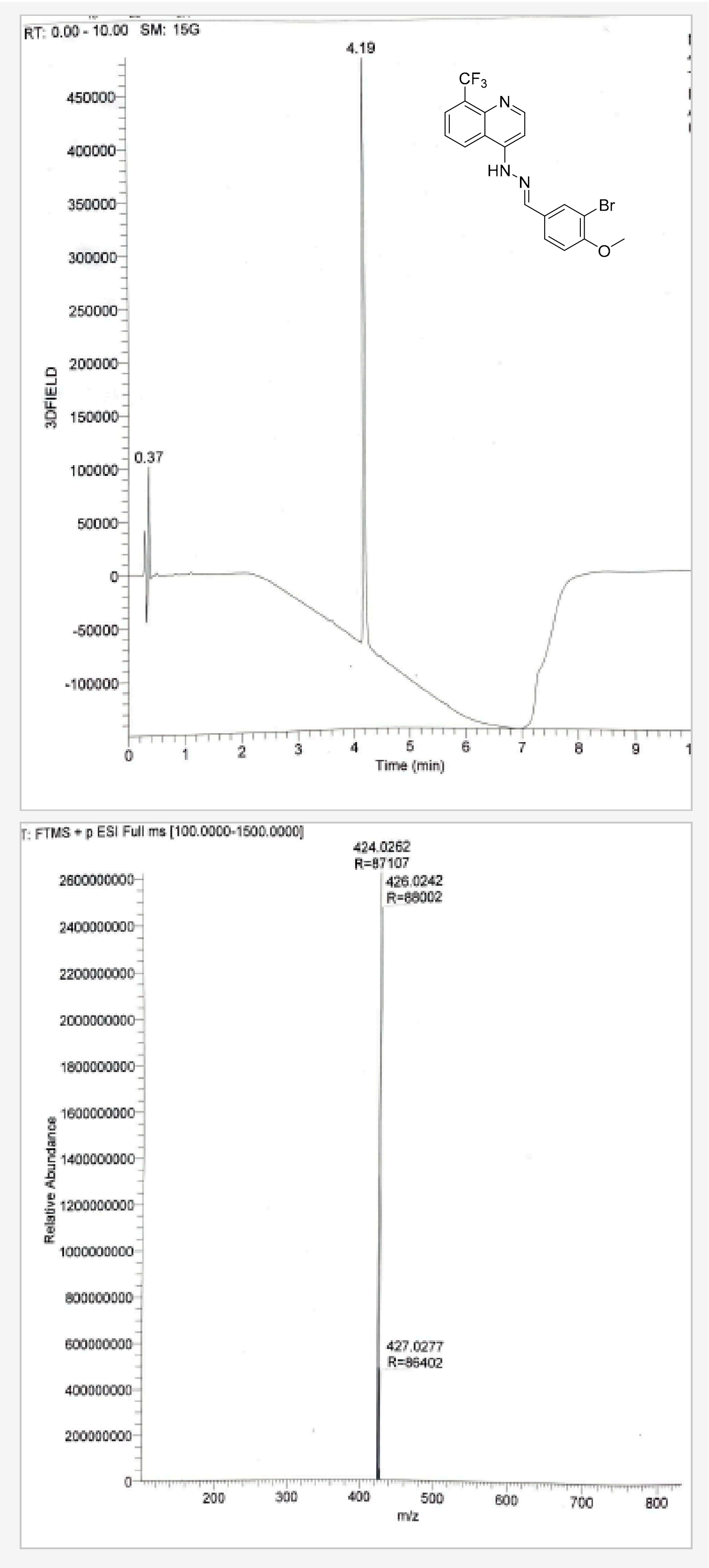

Figure S16. HRMS of (*E*)-4-(2-(3-bromo-4-methoxybenzylidene)hydrazinyl)-8-(trifluoromethyl)quinoline (**6d**).

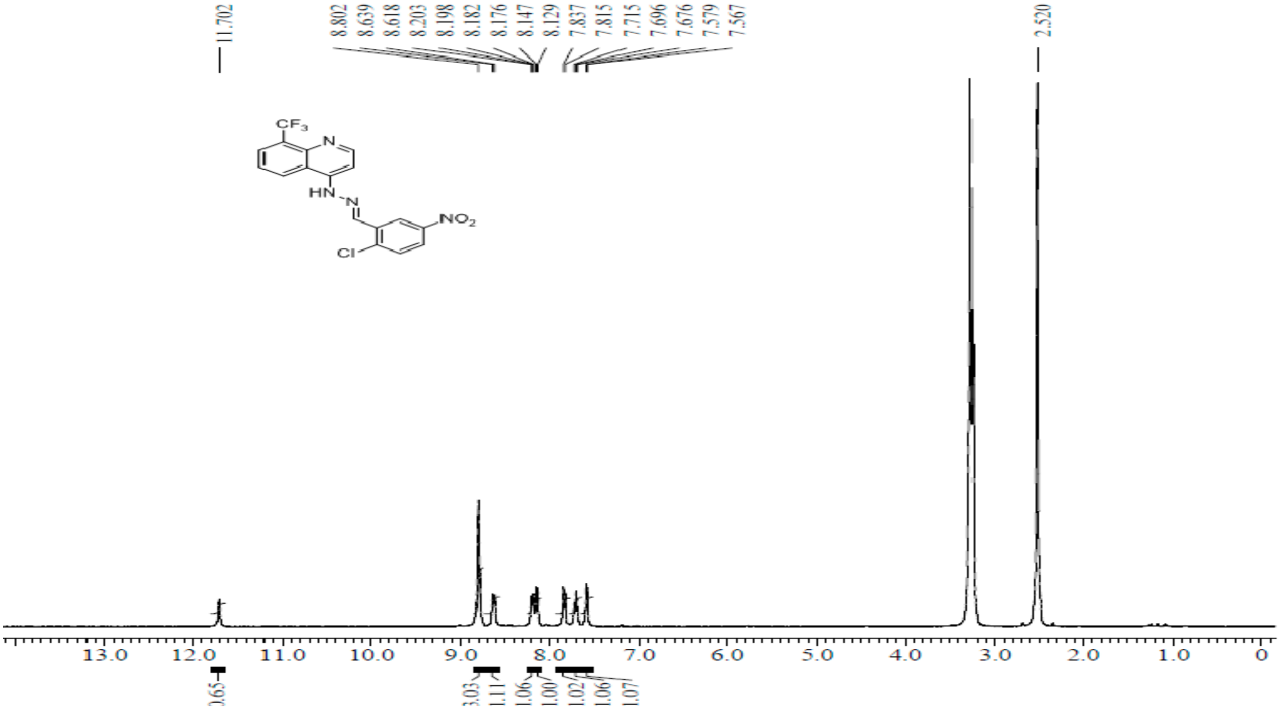

Figure S17. ^{1}H NMR spectrum of (*E*)-4-(2-(2-chloro-5-nitrobenzylidene)hydrazinyl)-8-(trifluoromethyl)quinoline (**6e**).

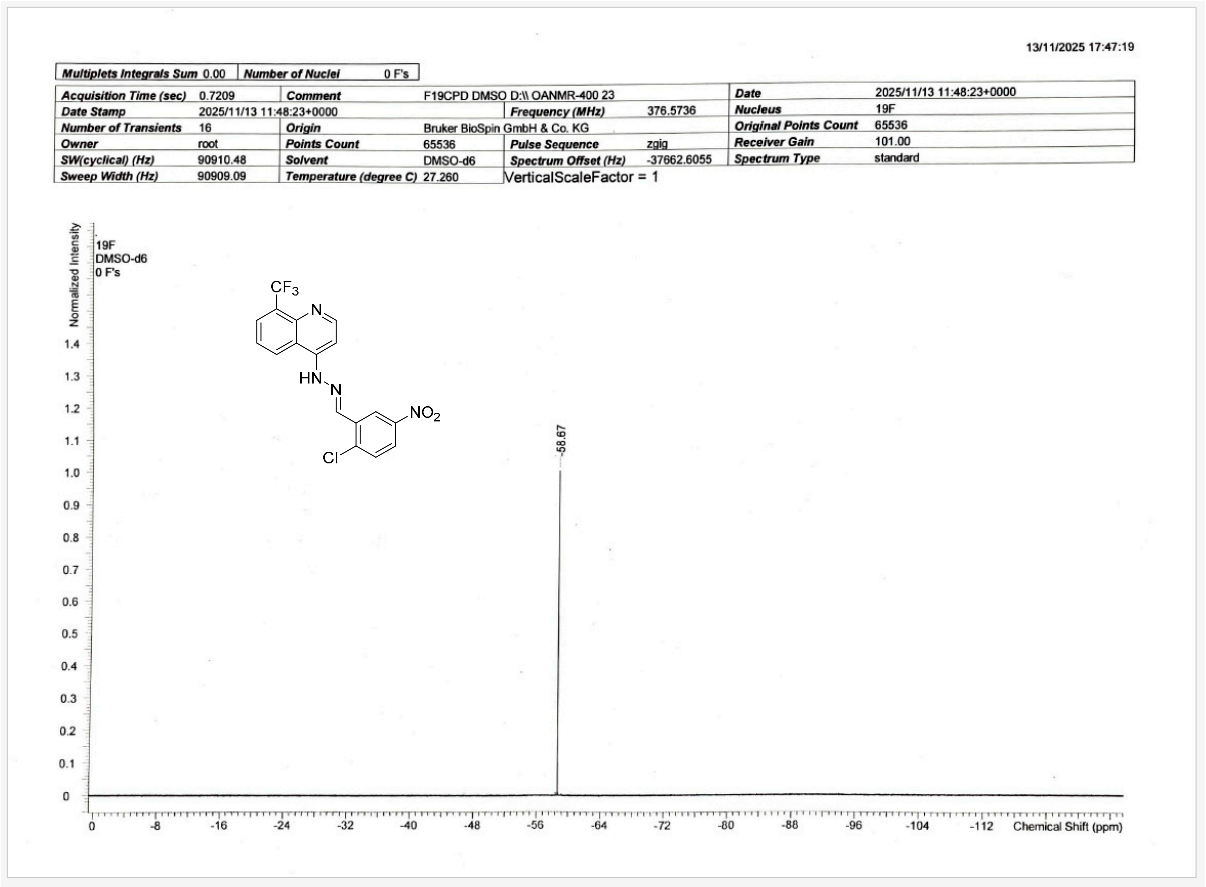

Figure S18. ^{19}F NMR spectrum of (E)-4-(2-(2-chloro-5-nitrobenzylidene) hydrazinyl) -8-(trifluoromethyl)quinoline (**6e**)

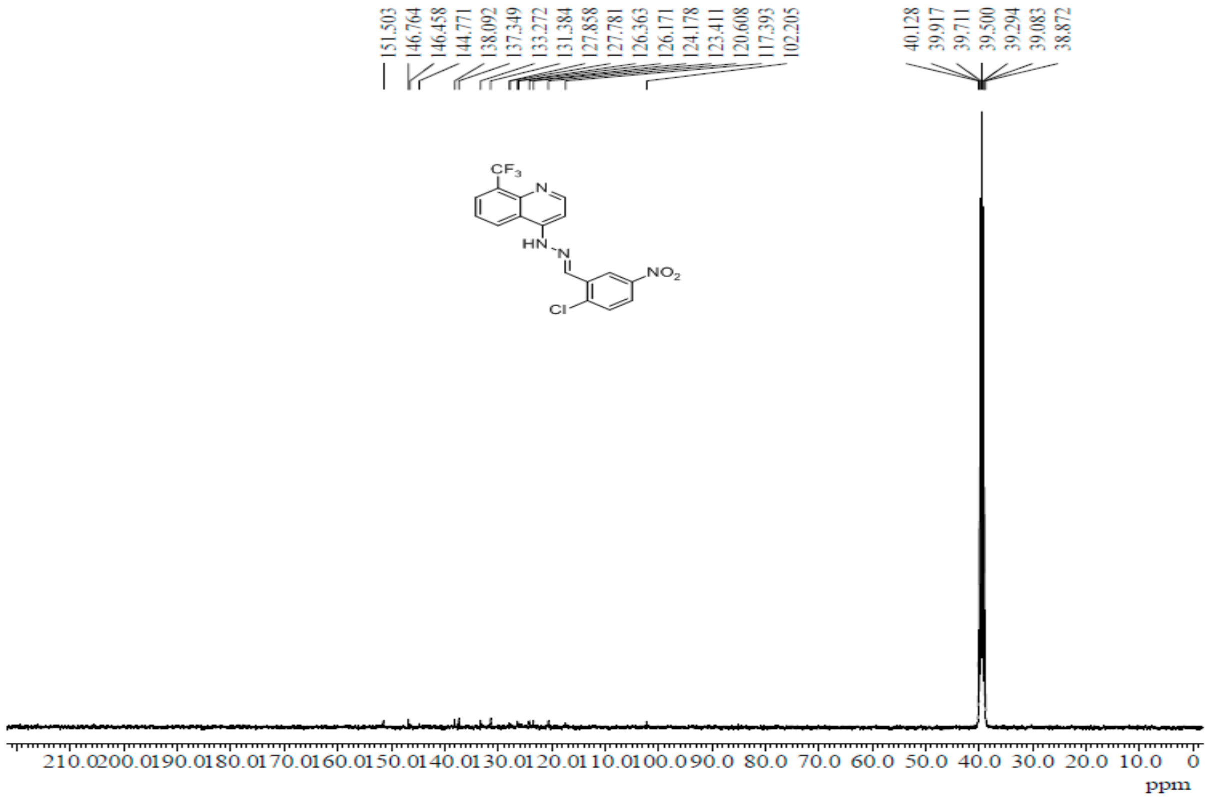

Figure S19. ^{13}C NMR spectrum of (*E*)-4-(2-(2-chloro-5-nitrobenzylidene)hydrazinyl)-8-(trifluoromethyl)quinoline (**6e**).

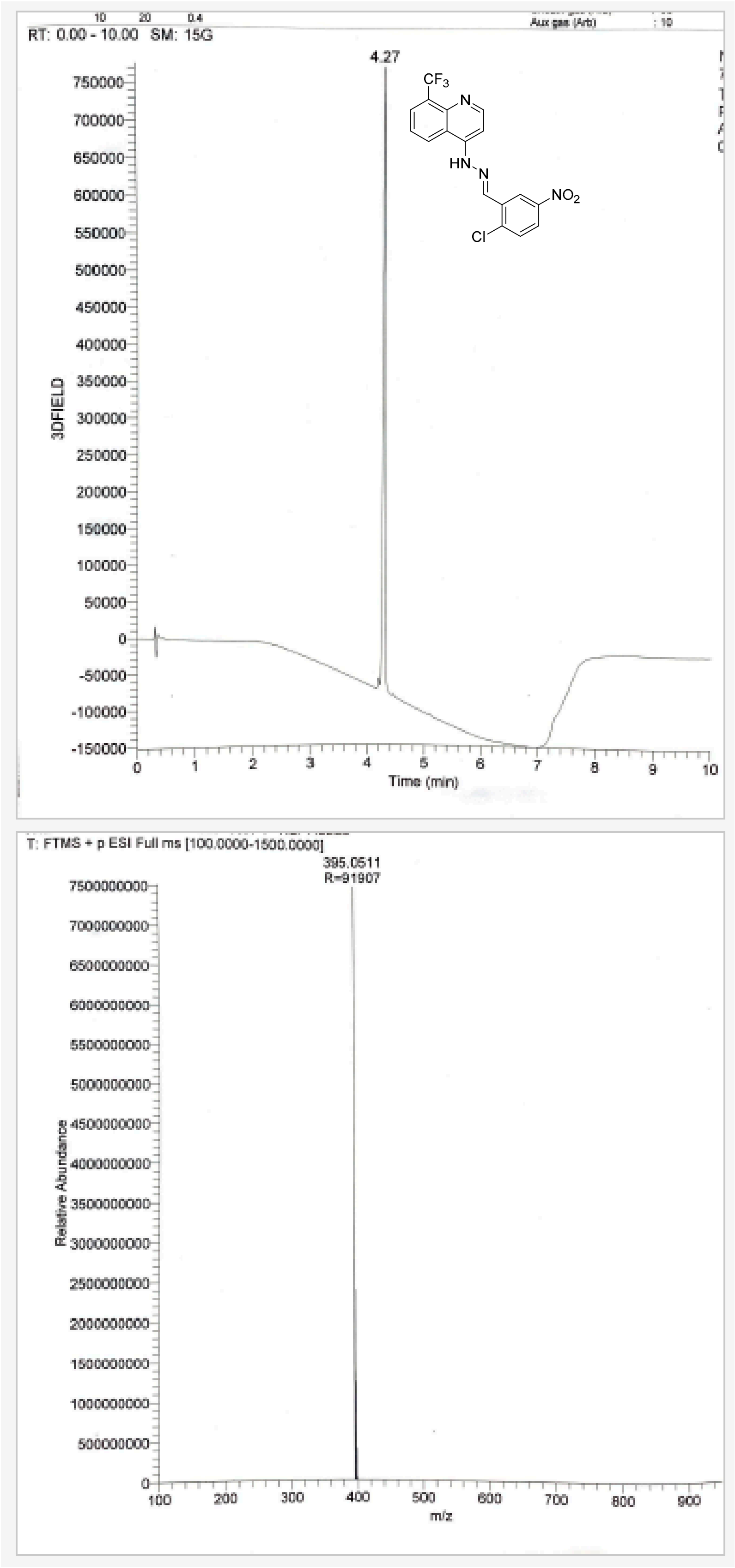

Figure S20. HRMS of (*E*)-4-(2-(2-chloro-5-nitrobenzylidene)hydrazinyl)-8-(trifluoromethyl)quinoline (**6e**).

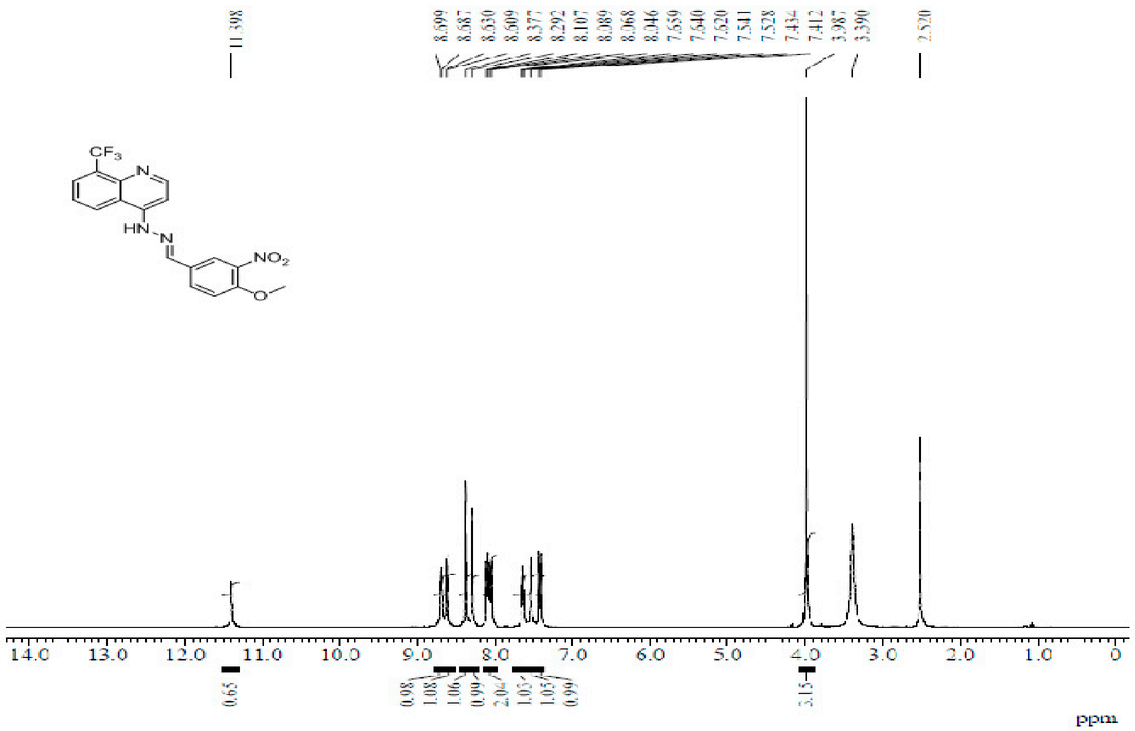

Figure S21. ^{1}H NMR spectrum of (*E*)-4-(2-(4-methoxy-3-nitrobenzylidene)hydrazinyl)-8-(trifluoromethyl)quinoline (**6f**).

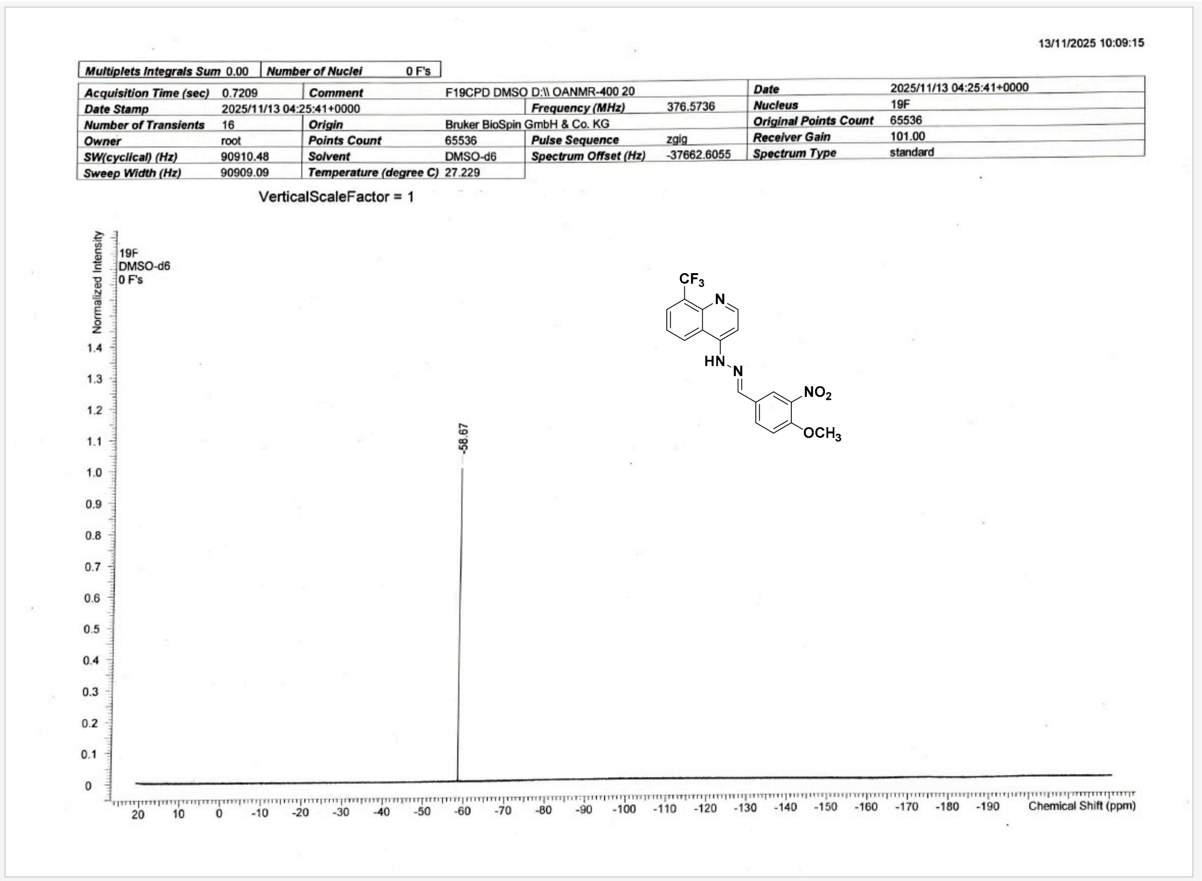

Figure S22. ^{19}F NMR spectrum of (*E*)-4-(2-(4-methoxy-3-nitrobenzylidene) hydrazinyl)-8-(trifluoromethyl)quinoline (**6f**)

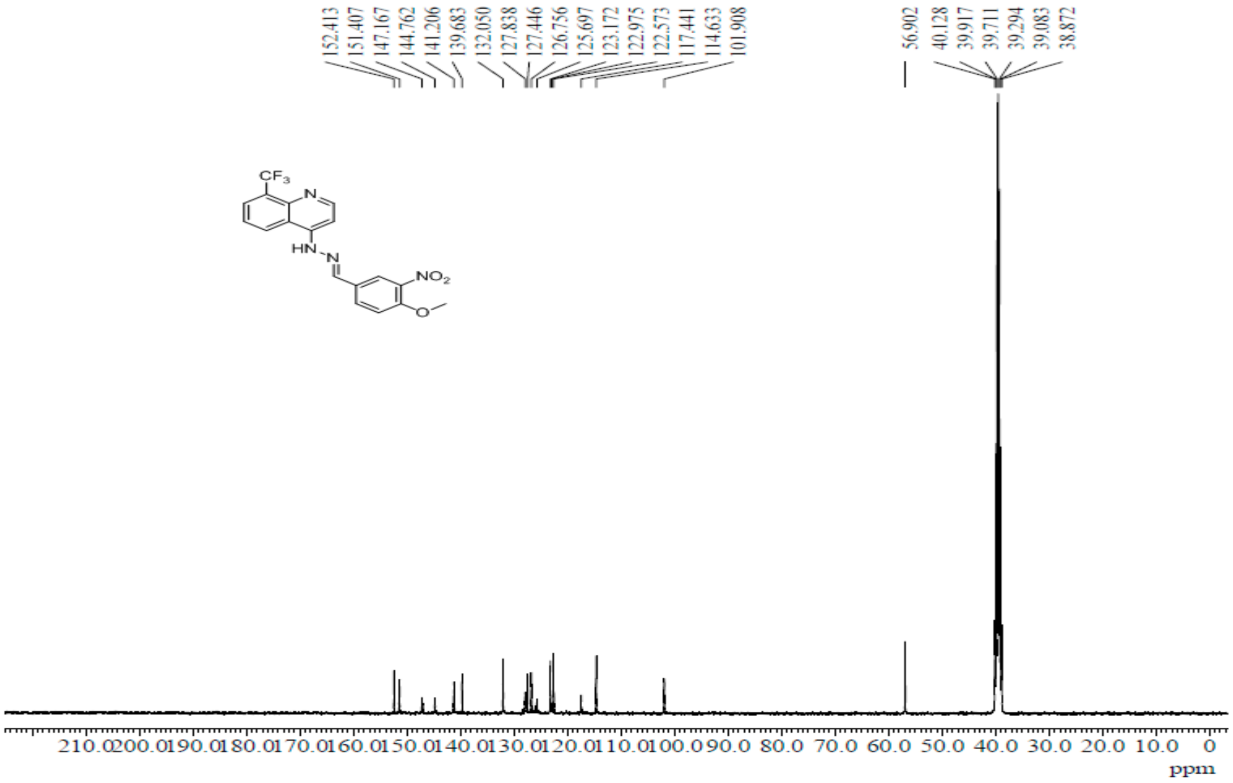

Figure S23. ^{13}C NMR spectrum of (*E*)-4-(2-(4-methoxy-3-nitrobenzylidene)hydrazinyl)-8-(trifluoromethyl)quinoline (**6f**).

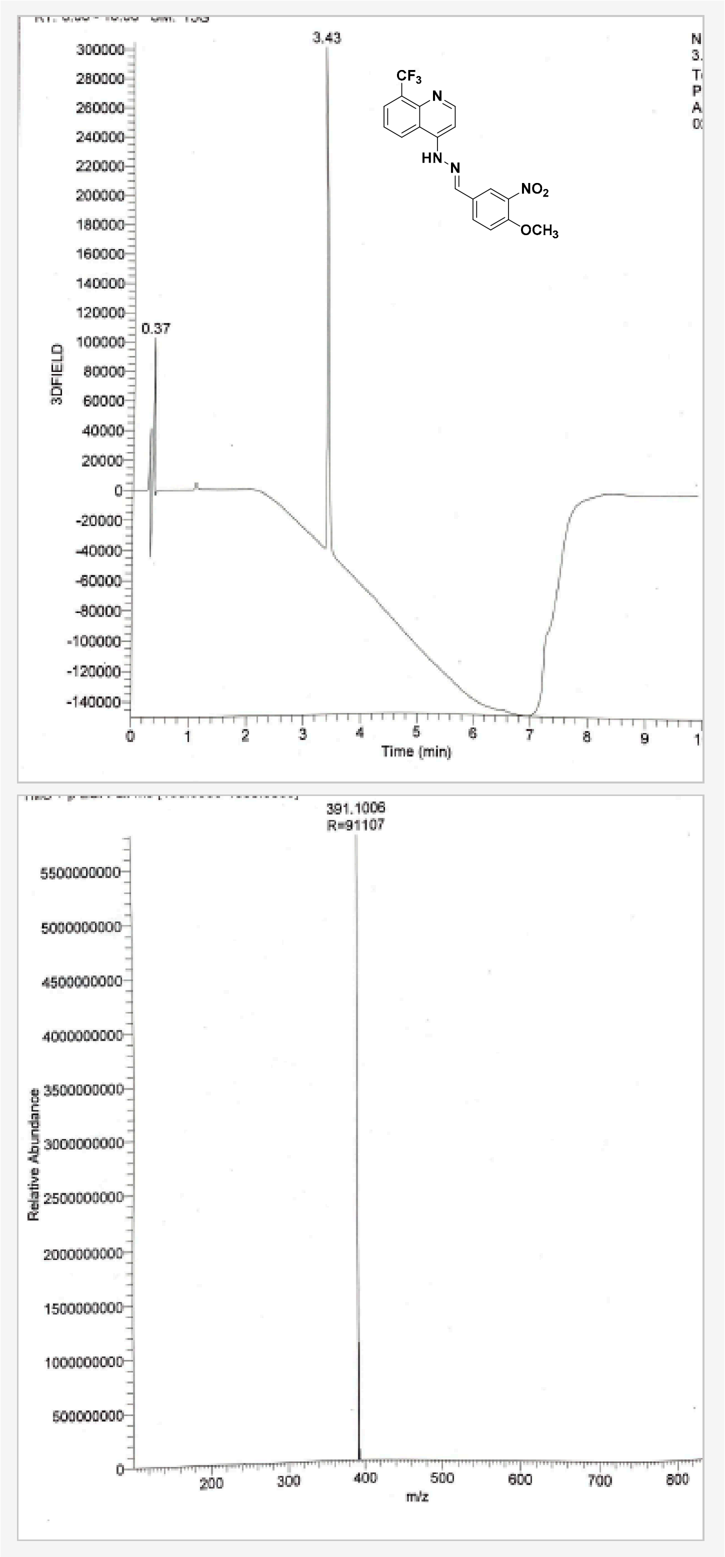

Figure S24. HRMS of (*E*)-4-(2-(4-methoxy-3-nitrobenzylidene)hydrazinyl)-8-(trifluoromethyl) quinoline (**6f**).

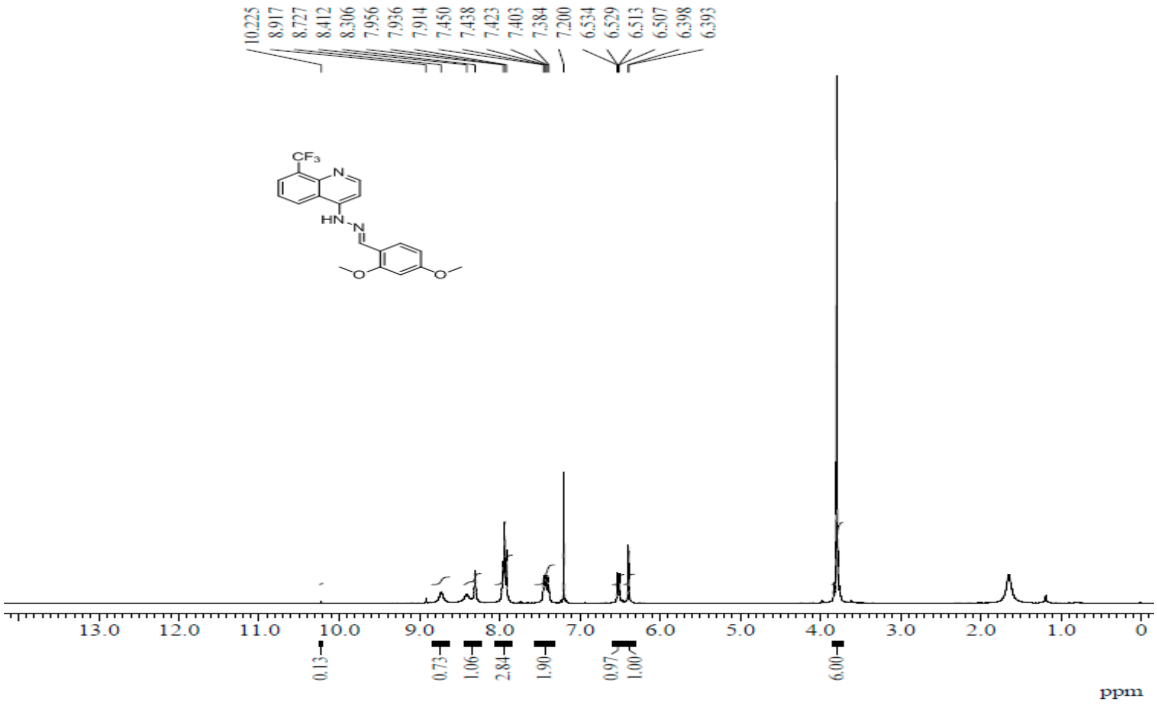

Figure S25. ^{1}H NMR spectrum of (*E*)-4-(2-(2,4-dimethoxybenzylidene)hydrazinyl)-8-(trifluoromethyl)quinoline (**6g**).

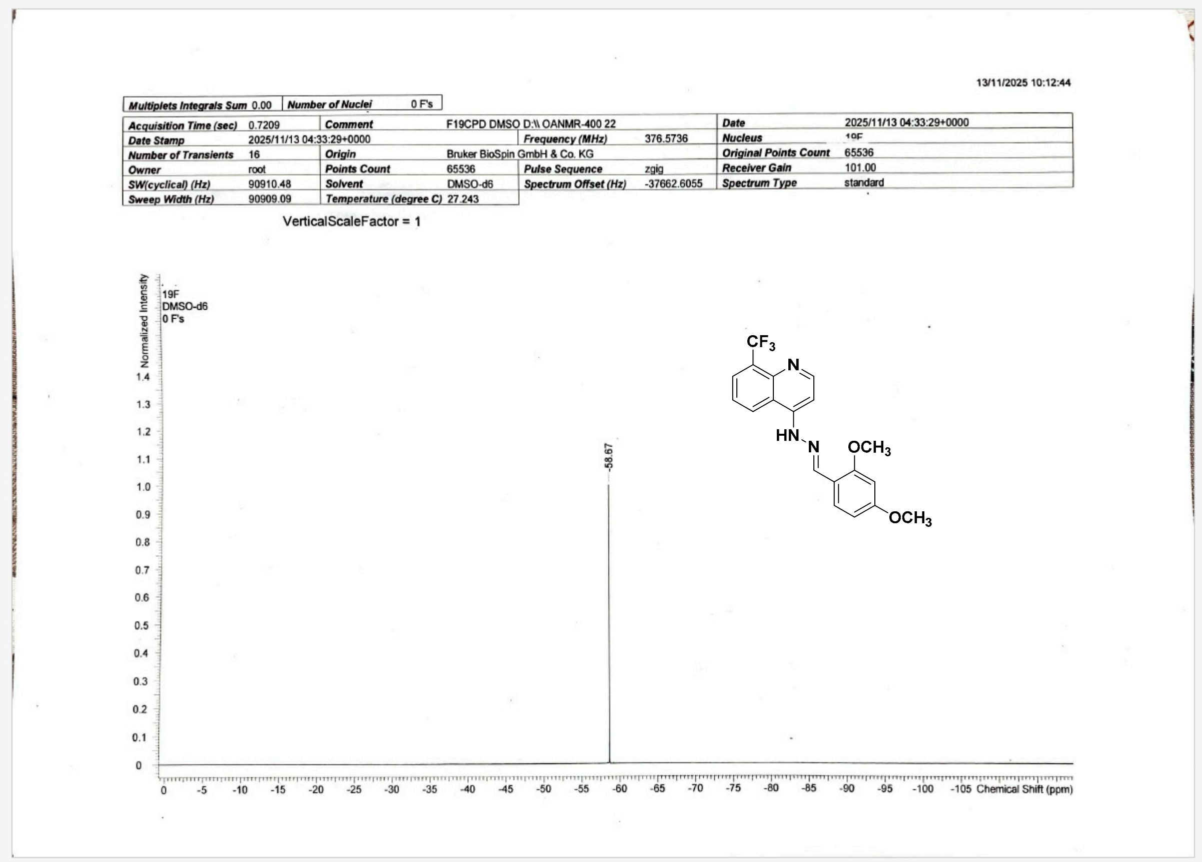

Figure S26. ^{19}F NMR spectrum of (*E*)-4-(2-(2,4-dimethoxybenzylidene) hydrazinyl)-8-(trifluoromethyl)quinoline (**6g**).

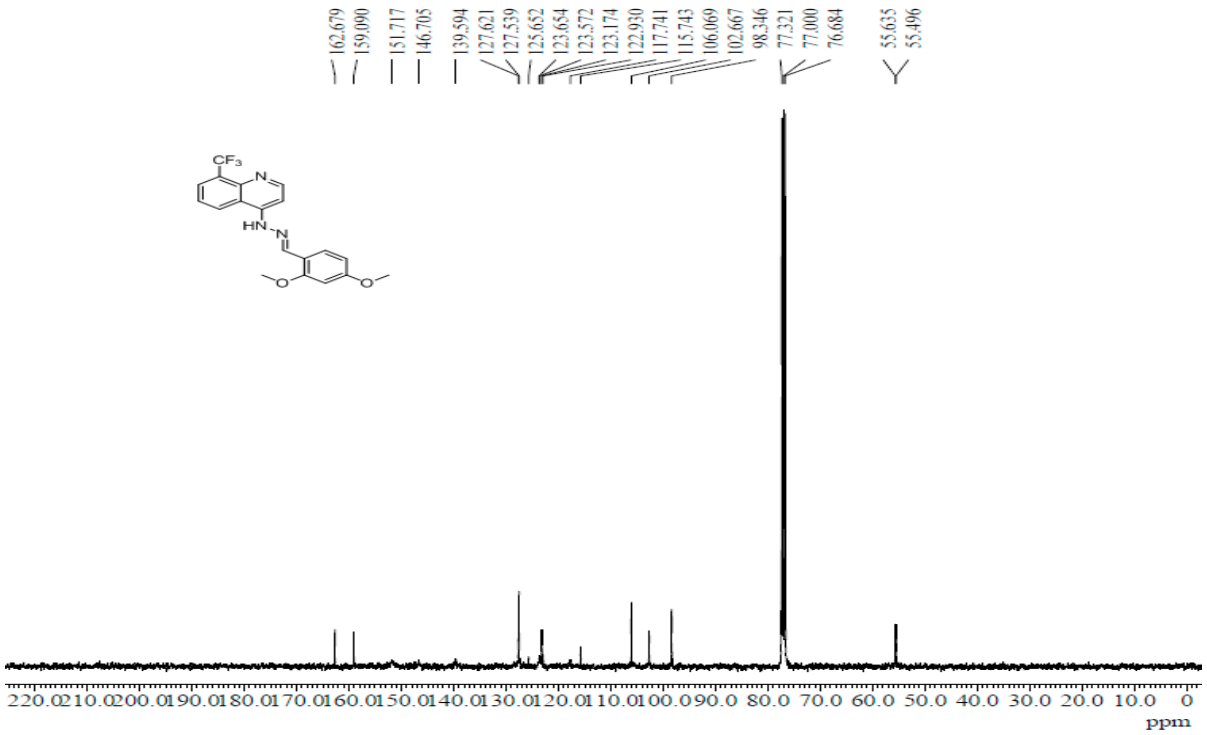

Figure S27. ^{13}C NMR spectrum of (*E*)-4-(2-(2,4-dimethoxybenzylidene)hydrazinyl)-8-(trifluoromethyl)quinoline (**6g**).

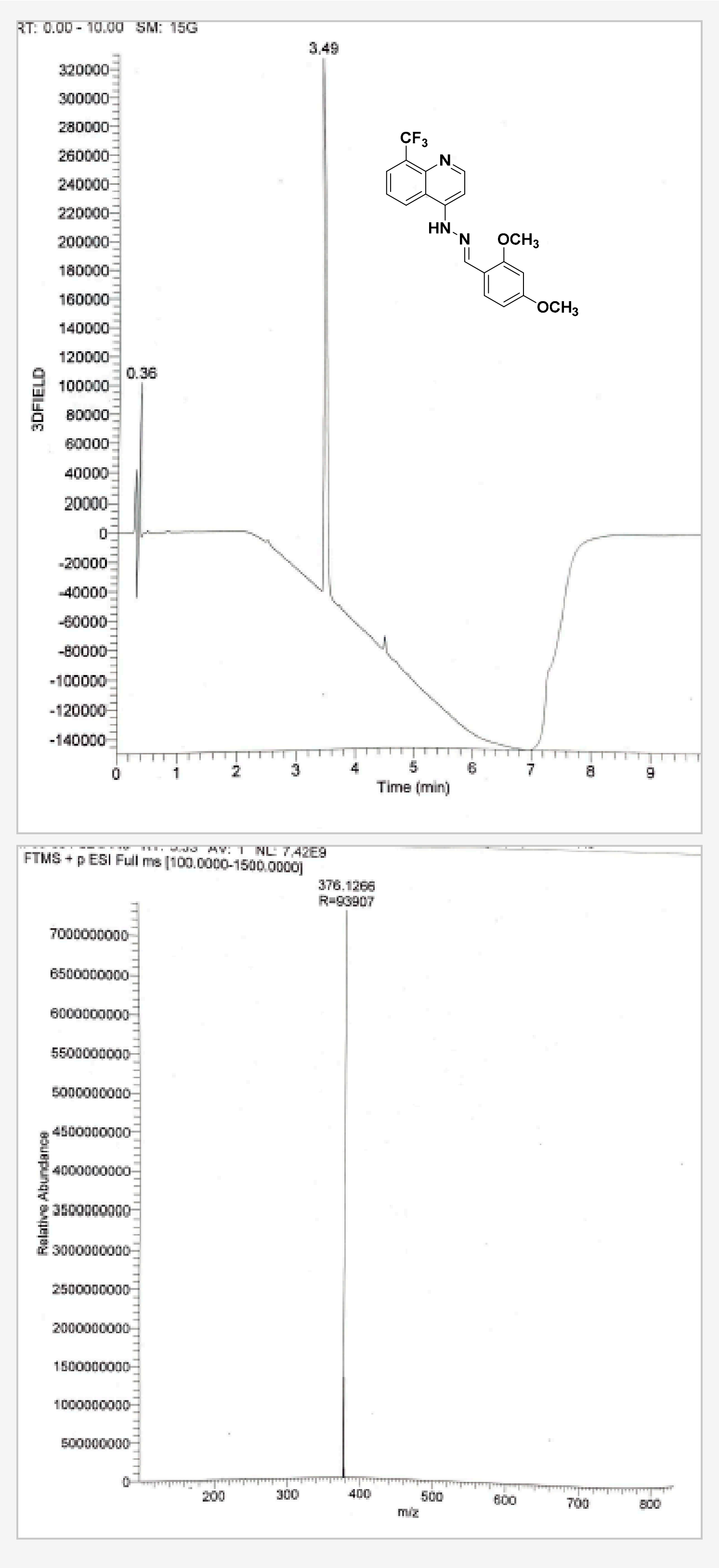

Figure S28. HRMS of (*E*)-4-(2-(2, 4-dimethoxybenzylidene) hydrazinyl)-8-(trifluoromethyl)quinoline (**6g**).

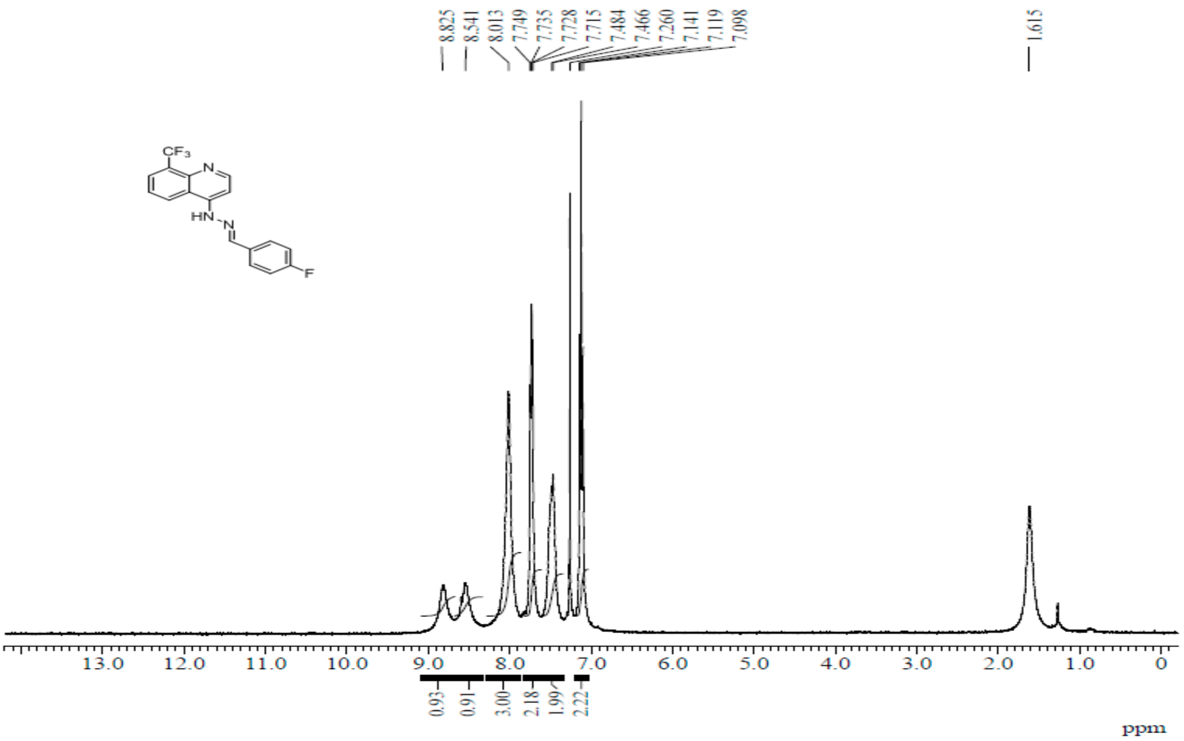

Figure S29. ^{1}H NMR spectrum of (*E*)-4-(2-(4-fluorobenzylidene)hydrazinyl)-8-(trifluoromethyl) quinoline (**6h**).

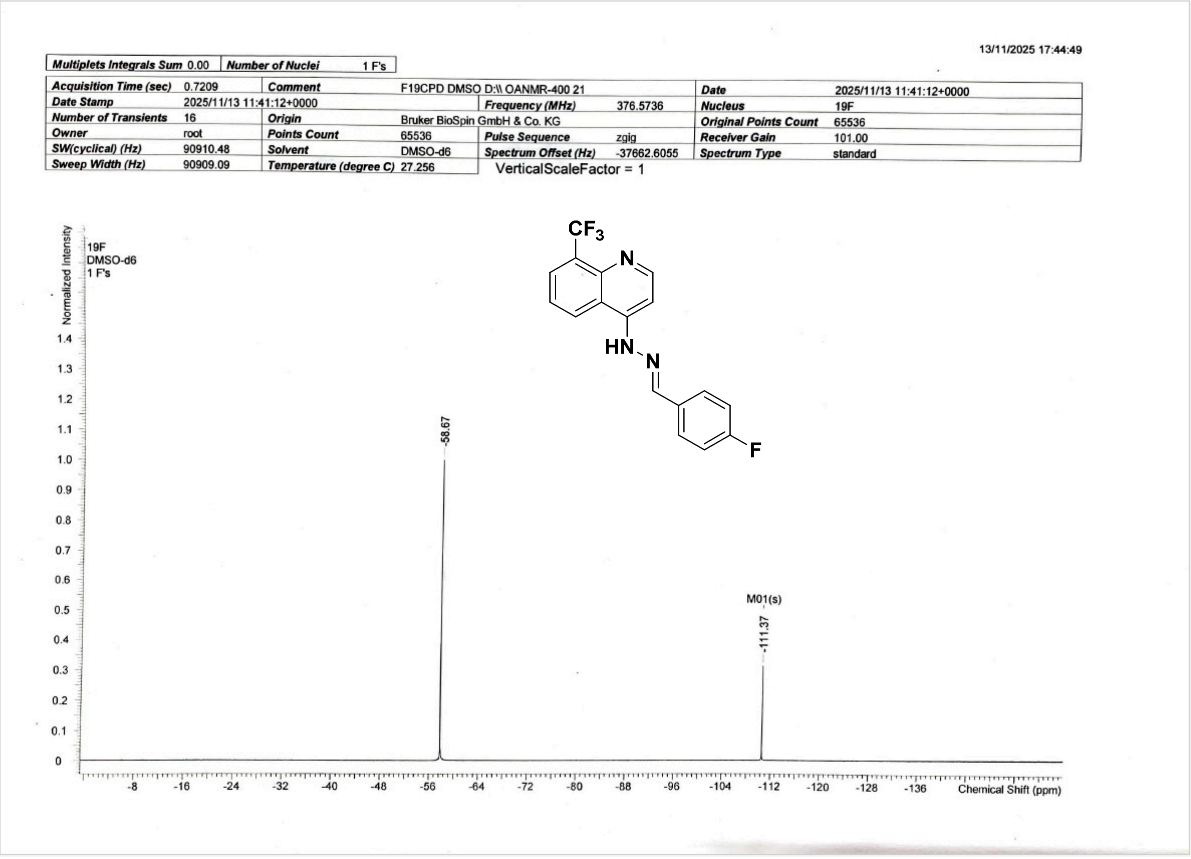

Figure S30. ^{19}F NMR spectrum of (*E*)-4-(2-(4-fluorobenzylidene)hydrazinyl)-8-(trifluoromethyl)quinoline (**6h**).

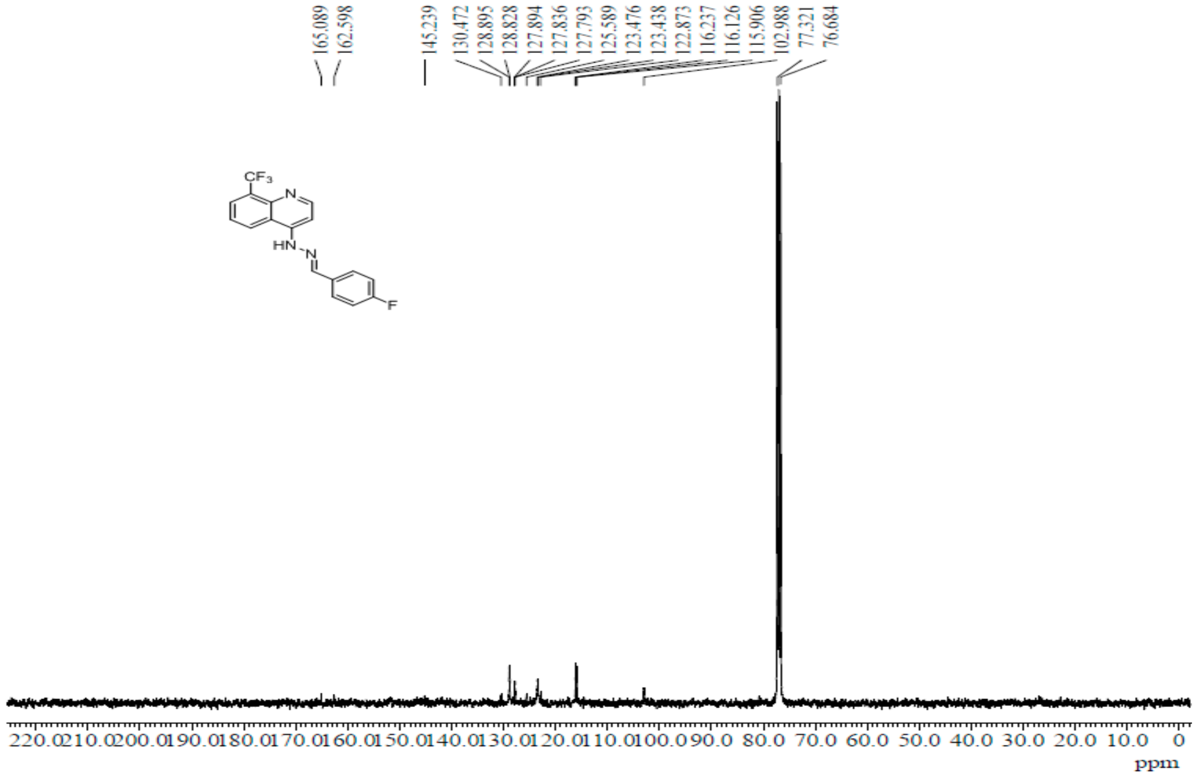

Figure S31. ^{13}C NMR spectrum of (*E*)-4-(2-(4-fluorobenzylidene)hydrazinyl)-8-(trifluoromethyl) quinoline (**6h**).

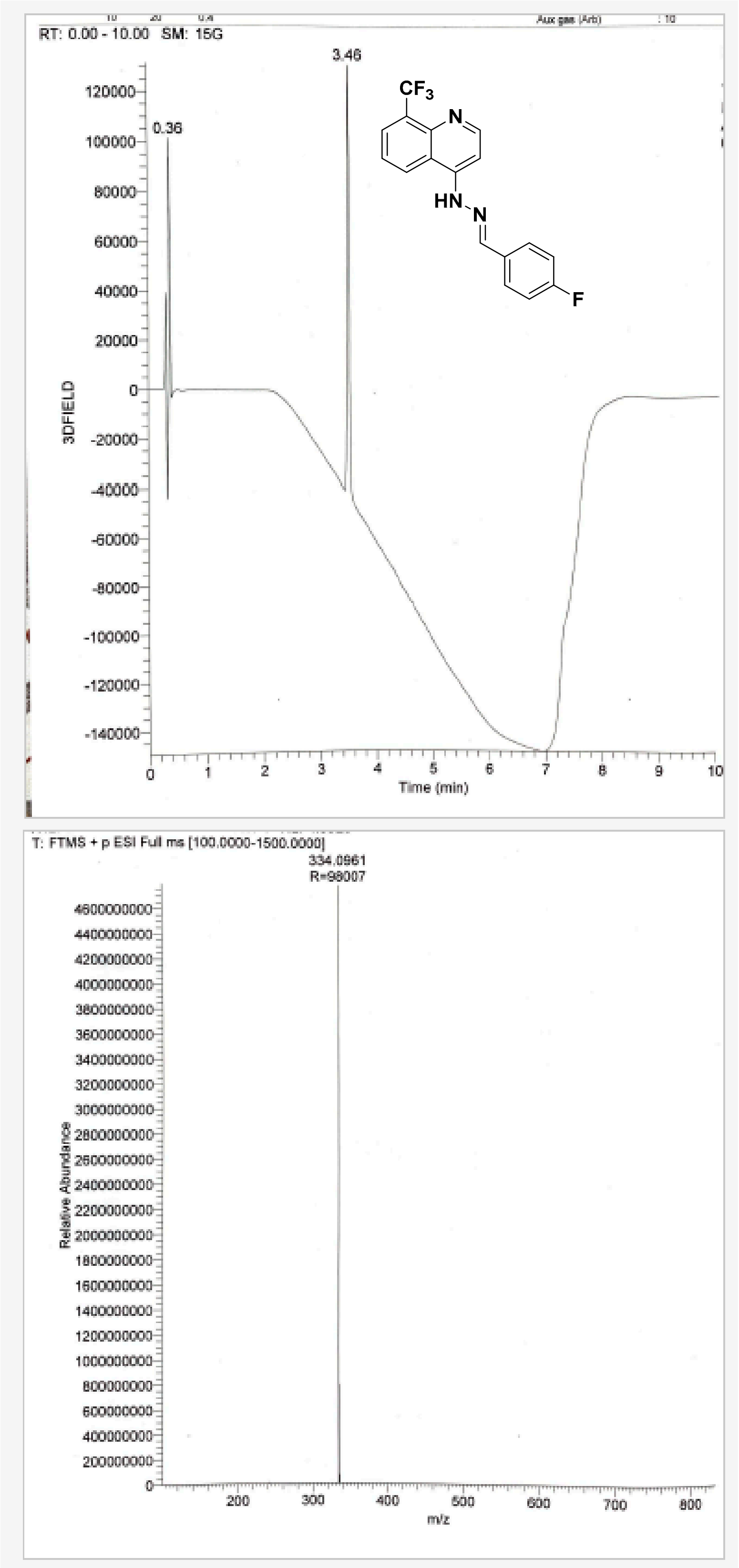

Figure S32. HRMS of (*E*)-4-(2-(4-fluorobenzylidene)hydrazinyl)-8-(trifluoromethyl)quinoline (**6h**).

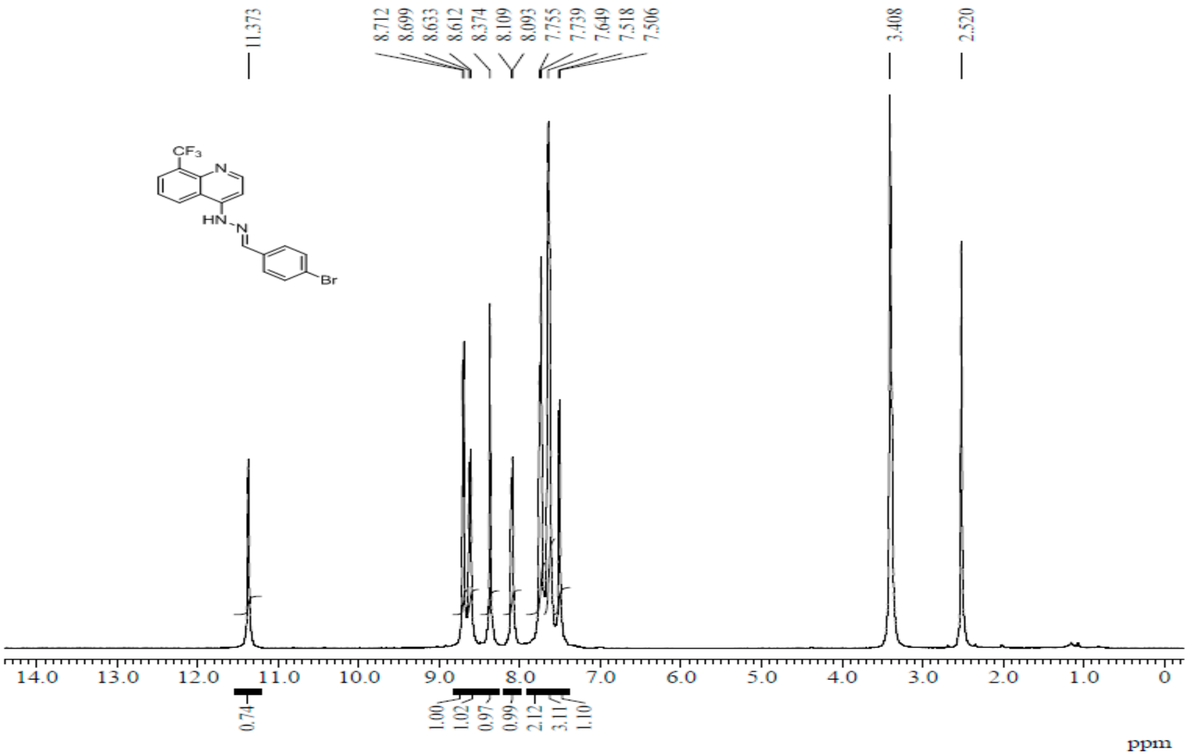

Figure S33. ^{1}H NMR spectrum of (*E*)-4-(2-(4-bromobenzylidene)hydrazinyl)-8-(trifluoromethyl) quinoline(**6i**).

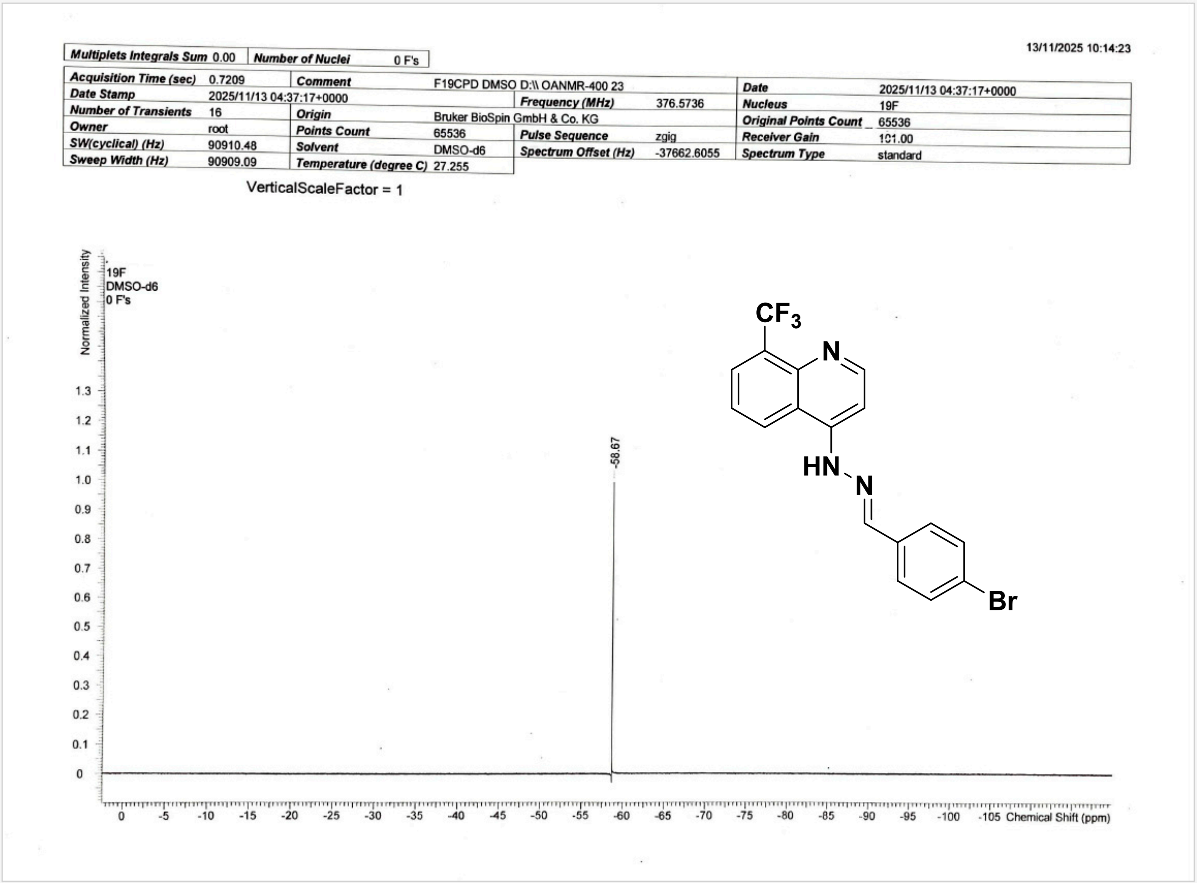

Figure S34. ^{19}F NMR spectrum of (*E*)-4-(2-(4-bromobenzylidene)hydrazinyl)-8-(trifluoromethyl)quinoline (**6i**).

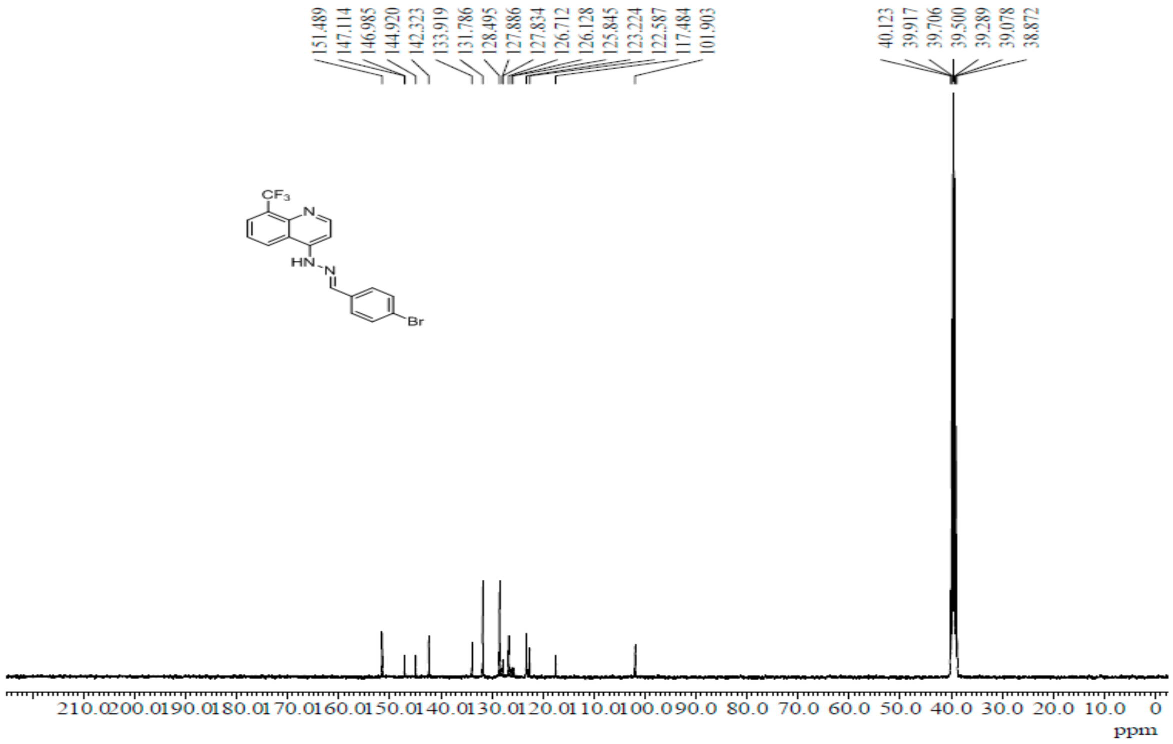

Figure S35. ^{13}C NMR spectrum of (*E*)-4-(2-(4-bromobenzylidene)hydrazinyl)-8-(trifluoromethyl) quinoline (**6i**).

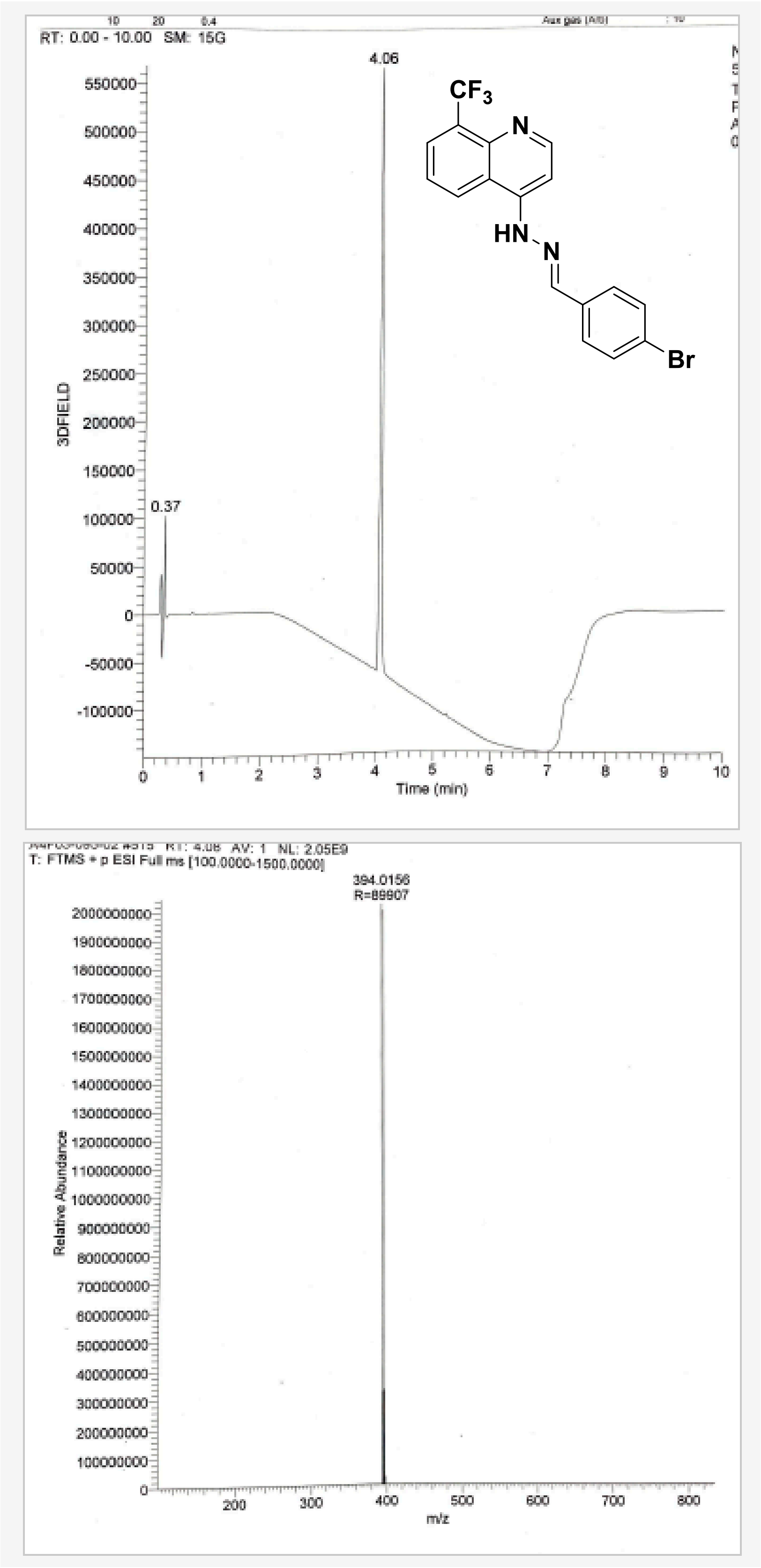

Figure S36. HRMS of (*E*)-4-(2-(4-bromobenzylidene)hydrazinyl)-8-(trifluoromethyl)quinoline (**6i**).

checkCIF/PLATON report

Structure factors have been supplied for datablock(s) 9d

THIS REPORT IS FOR GUIDANCE ONLY. IF USED AS PART OF A REVIEW PROCEDURE FOR PUBLICATION, IT SHOULD NOT REPLACE THE EXPERTISE OF AN EXPERIENCED CRYSTALLOGRAPHIC REFEREE.

No syntax errors found. CIF dictionary Interpreting this report

Datablock: 9d

Bond precision:	C-C = 0.0059 A		Wavelength=0.71073
Cell:	a=10.0716(7)	b=14.5234(8)	c=13.0461(7)
	alpha=90	beta=110.745(7)	gamma=90
Temperature:	293 K		

	Calculated	Reported
Volume	1784.6(2)	1784.6(2)
Space group	P 21/c	P 21/c
Hall group	-P 2ybc	-P 2ybc
Moiety formula	C18 H13 Br F3 N3 O	C18 H13 Br F3 N3 O
Sum formula	C18 H13 Br F3 N3 O	C18 H13 Br F3 N3 O
Mr	424.21	424.21
Dx,g cm-3	1.579	1.579
Z	4	4
Mu (mm-1)	2.345	2.345
F000	848.0	848.0
F000’	847.30	
h,k,lmax	11,17,15	11,17,15
Nref	3144	3144
Tmin,Tmax	0.514,0.611	0.514,0.611
Tmin’	0.502	

Correction method= # Reported T Limits: Tmin=0.514 Tmax=0.611
AbsCorr = NONE

Data completeness= 1.000 Theta(max)= 25.022

R(reflections)= 0.0493(2404) wR2(reflections)= 0.1439(3144)

S = 1.055 Npar= 237

```
PUBL010_ALERT_1_A  _publ_author_address is missing. Author(s) address(es).
PUBL012_ALERT_1_A  _publ_section_abstract is missing.
          Abstract of paper in English.
```

Alert level G

```
PUBL017_ALERT_1_G The _publ_section_references section is missing or
          empty.
```

```
  7 ALERT level A = Data missing that is essential or data in wrong format
  1 ALERT level G = General alerts. Data that may be required is missing
```

Publication of your CIF

You should attempt to resolve as many as possible of the alerts in all categories. Often the minor alerts point to easily fixed oversights, errors and omissions in your CIF or refinement strategy, so attention to these fine details can be worthwhile. In order to resolve some of the more serious problems it may be necessary to carry out additional measurements or structure refinements. However, the nature of your study may justify the reported deviations from journal submission requirements and the more serious of these should be commented upon in the discussion or experimental section of a paper or in the "special_details" fields of the CIF. *checkCIF* was carefully designed to identify outliers and unusual parameters, but every test has its limitations and alerts that are not important in a particular case may appear. Conversely, the absence of alerts does not guarantee there are no aspects of the results needing attention. It is up to the individual to critically assess their own results and, if necessary, seek expert advice.

If level A alerts remain, which you believe to be justified deviations, and you intend to submit this CIF for publication in a journal, you should additionally insert an explanation in your CIF using the Validation Reply Form (VRF) below. This will allow your explanation to be considered as part of the review process.

Validation response form

Please find below a validation response form (VRF) that can be filled in and pasted into your CIF.

```
# start Validation Reply Form
_vrf_PUBL004_GLOBAL
;
PROBLEM: The contact author's name and address are missing,
RESPONSE: ...
;
_vrf_PUBL005_GLOBAL
;
PROBLEM: _publ_contact_author_email, _publ_contact_author_fax and
RESPONSE: ...
;
_vrf_PUBL006_GLOBAL
;
PROBLEM: _publ_requested_journal is missing
RESPONSE: ...
```

checkCIF

```
;
_vrf_PUBL008_GLOBAL
;
PROBLEM: _publ_section_title is missing. Title of paper.
RESPONSE: ...
;
_vrf_PUBL009_GLOBAL
;
PROBLEM: _publ_author_name is missing. List of author(s) name(s).
RESPONSE: ...
;
_vrf_PUBL010_GLOBAL
;
PROBLEM: _publ_author_address is missing. Author(s) address(es).
RESPONSE: ...
;
_vrf_PUBL012_GLOBAL
;
PROBLEM: _publ_section_abstract is missing.
RESPONSE: ...
;
# end Validation Reply Form
```

If you wish to submit your CIF for publication in Acta Crystallographica Section C or E, you should upload your CIF via the web. If you wish to submit your CIF for publication in IUCrData you should upload your CIF via the web. If your CIF is to form part of a submission to another IUCr journal, you will be asked, either during electronic submission or by the Co-editor handling your paper, to upload your CIF via our web site.

PLATON version of 18/05/2022; check.def file version of 19/01/2022

utablock 9d - ellipsoid plot

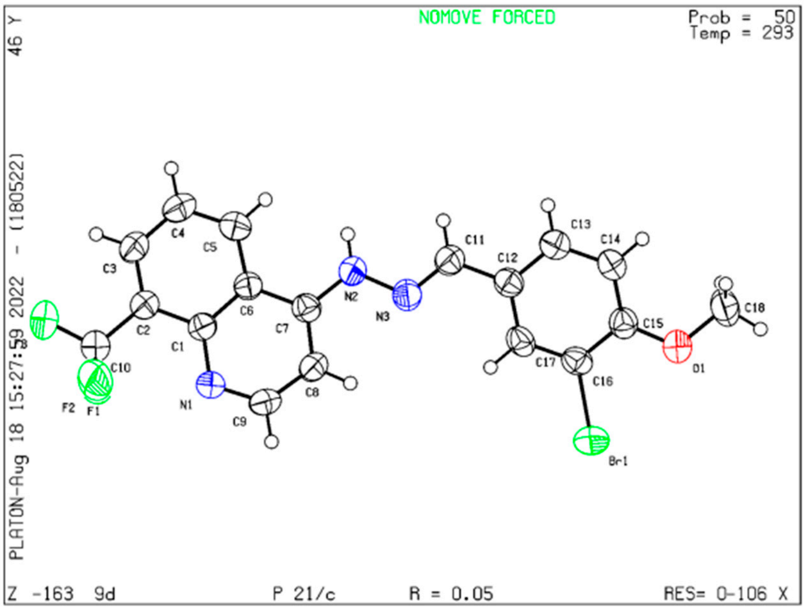

Figure S37. Check CIF report on (*E*)-4-(2-(3-bromo-4-methoxybenzylidene)hydrazinyl)-8-(trifluoromethyl)quinoline (**6d**)

Table S1. Tabular presentation of in silico ADME parameters for all compounds (**6a-6i**) under study.

	**Properties**		**Units**	**Compounds Code**								
				**6a**	**6b**	**6c**	**6d**	**6e**	**6f**	**6g**	**6h**	**6i**
**ADME PROFILE**	**Physiochemical parameters**	**Formula**		$C_{17}H_{10}BrF_4N_3$	$C_{18}H_{13}BrF_3N_3O$	$C_{19}H_{15}F_3N_4O_4$	$C_{18}H_{13}BrF_3N_3O$	$C_{17}H_{10}ClF_3N_4O_2$	$C_{18}H_{13}F_3N_4O_3$	$C_{19}H_{16}F_3N_3O_2$	$C_{17}H_{11}F_4N_3$	$C_{17}H_{11}BrF_3N_3$
		**Molecular weight**	g/mol	412.18	424.21	420.34	424.21	394.74	390.32	375.34	333.28	394.19
		**Mol. refractivity**		91.88	98.41	106.03	98.41	98.05	99.54	97.21	84.18	91.92
		**TPSA**	Å²	37.28	46.51	101.56	46.51	83.10	92.33	55.74	37.28	37.28
	**Lipophilicity**	**ILOGP**		3.00	3.41	2.59	3.14	2.29	2.31	3.39	2.72	3.05
		**SILICOS-IT**		5.68	5.31	2.57	5.31	3.08	2.49	4.70	5.00	5.25
	**Water Solubility**	**Log S (ESOL), Class**		-6.06	-5.97	-6.79	-6.08	-5.64	-5.12	-5.13	-5.15	-5.90
	**Pharmaco-kinetics**	**Intestinal absorption (human)**	%	88.23	88.368	95.172	88.424	86.117	89.835	91.9	88.893	87.924
		**VDss (human)**	log L/kg	0.168	0.143	-0.059	0.063	-0.025	-0.131	0.236	-0.059	0.13
		**Fraction unbound (human)**	Fu	0.058	0.038	0	0.021	0	0	0.03	0.042	0.018
		**CNS permeability**	log PS	-1.257	-1.193	-2.132	-1.11	-1.603	-1.918	-1.949	-1.157	-1.131
		**BBB permeant**	Yes/No	No	No	No	No	No	No	No	No	No
		**Log K_p(skin perm.)**	cm/s	-4.92	-5.09	-5.70	-5.09	-5.05	-5.49	-5.30	-4.93	-4.88
		**CYP1A2**	Yes/No	Yes	Yes	Yes	Yes	Yes	Yes	Yes	Yes	Yes
		**CYP2D6**	Yes/No	No	No	Yes	No	No	No	Yes	Yes	No
	**Drug-likeness Rules**	**Lipinski (Pfizer)**	Yes/No	Yes	Yes	Yes	Yes	Yes	Yes	Yes	Yes	Yes
		**Ghose (Amgen)**	Yes/No	No	No	Yes	No	No	Yes	No	No	No
		**Veber (GSK)**	Yes/No	Yes	Yes	Yes	Yes	Yes	Yes	Yes	Yes	Yes
		**Egan (Pharmacia)**	Yes/No	No	No	Yes	No	No	Yes	Yes	No	No
		**Muege (Bayer)**	Yes/No	No	No	Yes	No	No	Yes	Yes	Yes	No
		**Bioavailability Score**		0.55	0.55	0.55	0.55	0.55	0.55	0.55	0.55	0.55
	**Medicinal Chemistry**	**PAINS**	alert	0	0	0	0	0	0	0	0	0
		**Brenk**	alert	1	1	3	1	3	3	1	1	1
		**Synthetic accessibility**	1: very easy 10: very difficult	2.78	2.88	3.24	2.76	2.92	2.98	2.96	2.64	2.67
	**Excretion**	**Total Clearance**	(log ml/min/kg)	0.134	0.406	-0.081	0.253	0.412	0.133	0.19	-0.158	0.251
		**Renal OCT2 substrate**	Yes/No	No	No	No	Yes	No	No	No	No	No
